# LOV1 protein of *Pseudomonas cichorii* JBC1 modulates its virulence and lifestyles in response to blue light

**DOI:** 10.1038/s41598-024-66422-1

**Published:** 2024-07-08

**Authors:** Nguyen Van Khanh, Yong Hoon Lee

**Affiliations:** 1 Division of Biotechnology, Jeonbuk National University, 79 Gobong-ro, Iksan-si, Jeollabuk-do 54596 Republic of Korea; 2 Advanced Institute of Environment and Bioscience, Plant Medical Research Center, and Institute of Bio-industry, Jeonbuk National University, Jeonju-si, Republic of Korea

**Keywords:** Bacterial photoreceptors, LOV, *Pseudomonas cichorii* JBC1, Virulence, Microbiology, Plant sciences

## Abstract

Bacteria perceive light signals via photoreceptors and modulate many physiological and genetic processes. The impacts played by light, oxygen, or voltage (LOV) and blue light (BL) photosensory proteins on the virulence-related traits of plant bacterial pathogens are diverse and complex. In this study, we identified LOV protein (Pc-LOV1) from *Pseudomonas cichorii* JBC1 (PcJBC1) and characterized its function using LOV1-deficient mutant (JBC1^Δlov1^). In the dark state, the recombinant Pc-LOV1 protein showed an absorption band in UV-A region with a double peak at 340 nm and 365 nm, and within the blue-region, it exhibited a main absorption at 448 nm along with two shoulder peaks at 425 nm and 475 nm, which is a typical feature of oxidized flavin within LOV domain. The adduct-state lifetime (τ_rec_) of Pc-LOV1 was 67.03 ± 4.34 min at 25 °C. BL negatively influenced the virulence of PcJBC1 and the virulence of JBC1^Δlov1^ increased irrespective of BL, indicating that Pc-LOV1 negatively regulates PcJBC1 virulence. Pc-LOV1 and BL positively regulated traits relevant to colonization on plant surface, such as adhesion to the plant tissue and biofilm formation. In contrast, swarming motility, exopolysaccharide production, and siderophore synthesis were negatively controlled. Gene expression supported the modulation of bacterial features by Pc-LOV1. Overall, our results suggest that the LOV photosensory system plays crucial roles in the adaptive responses and virulence of the bacterial pathogen PcJBC1. The roles of other photoreceptors, sensing of other wavelengths, and signal networking require further investigation.

## Introduction

Bacteria respond to light by employing numerous photosensory proteins conserved in their genome and regulate physiological and ecological processes via intercellular downstream signal transduction for successful adaptation to the environment^1–4^. Photosensory proteins containing light, oxygen, or voltage (LOV) domains are widespread in multiple kingdoms including bacteria, archaea, fungi, algae, and plants^5^. They play roles in controlling many cellular responses such as virulence, gene expression, stress responses, photosynthesis pigment synthesis, and regulation of circadian rhythms^6–8^. LOV proteins respond to blue light (BL) through conformational changes that result from light-mediated protein–flavin adduct formation at a conserved cysteine in the LOV domain^9^, which can subsequently activate various C-terminal effector domains^10^. Swartz et al.^9^ reported that the prokaryotes *Brucella melitensis*, *Brucella abortus, Erythrobacter litoralis,* and *Pseudomonas syringae* possess flavin mononucleotide (FMN)-binding histidine kinases (HKs)-containing LOV domains. Characterization of LOV-HK from *Pseudomonas syringae* pv. *tomato* DC3000 (PstDC3000) showed that the LOV protein associates primarily with an FMN and secondarily with a flavin adenine dinucleotide (FAD) and exhibits BL-mediated autophosphorylation^11^.

Pathogenic bacteria utilized light to increase their virulence and access their location to enter host plants^12^. Virulence of *Xanthomonas axonopodis* pv. *citri* strain 306 (Xac306) were regulated by light via LOV-HK protein^1^. The LOV domain photoreceptor of PstDC3000 showed attenuated virulence when exposed to light in *Arabidopsis* leaves^13^. Ricci et al.^14^ demonstrated that blue- and red-light photoreceptors rendered PstDC3000 less virulent and less invasive in tomato plants. The infection of macrophages by *B. abortus* was stimulated by light^9^.

Light also regulated ecological characteristics such as the motility and attachment of pathogenic bacteria as well as growth. LOV-HK protein reduced the attachment of *Rhizobium leguminosarum* to abiotic surfaces^15^, whereas the intercellular attachment of *Caulobacter crescentus* was increased^16^. LOV-HK of Xac modulated its physiological attributes to mediate host plant colonization^1^. LOV proteins of *P. syringae* pv. *syringae* B278a (PssB278a) positively regulated swarming motility^4^. LOV influenced the motility and attachment of PstDC3000 depending on the light wavelengths^17^.

*Pseudomonas cichorii* JBC1 (PcJBC1) infects a wide range of plant hosts^18,19^. Losi and Gärtner^2^ reported that PcJBC1 contains a photosensory blue-light-sensing LOV protein, as well as red- and far-red-light-sensing BphP. However, the roles of PcJBC1 photoreceptors in the physiology, lifestyle decisions, and virulence of the pathogen are yet to be explored.

In this study, we scrutinized the PcJBC1 genome and detected a putative photosensory LOV-HK-like protein (Pc-LOV1) by analyzing its domain architecture using a conserved domain database (CDD). To investigate the role of Pc-LOV1 in PcJBC1, we constructed a mutant strain lacking the functional *lov1* gene and investigated the effect of Pc-LOV1 on the virulence of PcJBC1 under BL-illuminated and dark conditions. The effects of the Pc-LOV1 protein on biological attributes relevant to infection processes, such as motility, adhesion, and biofilm formation, were also assessed. We further studied the influence of Pc-LOV1 on the production and secretion of metabolites important for the survival and colonization of PcJBC1 in plant tissues. The recombinant Pc-LOV1 protein was overexpressed in *Escherichia coli* and the UV–Vis absorption spectra of the purified protein were analyzed. Taken together, the results of this study indicate that Pc-LOV1 plays a crucial role in the pathological features of PcJBC1 in response to BL. To our knowledge, this is the first report on the functional roles of photoreceptors in *P. cichorii*.

## Results

### Domain architecture of Pc-LOV1

**Figure 1:**
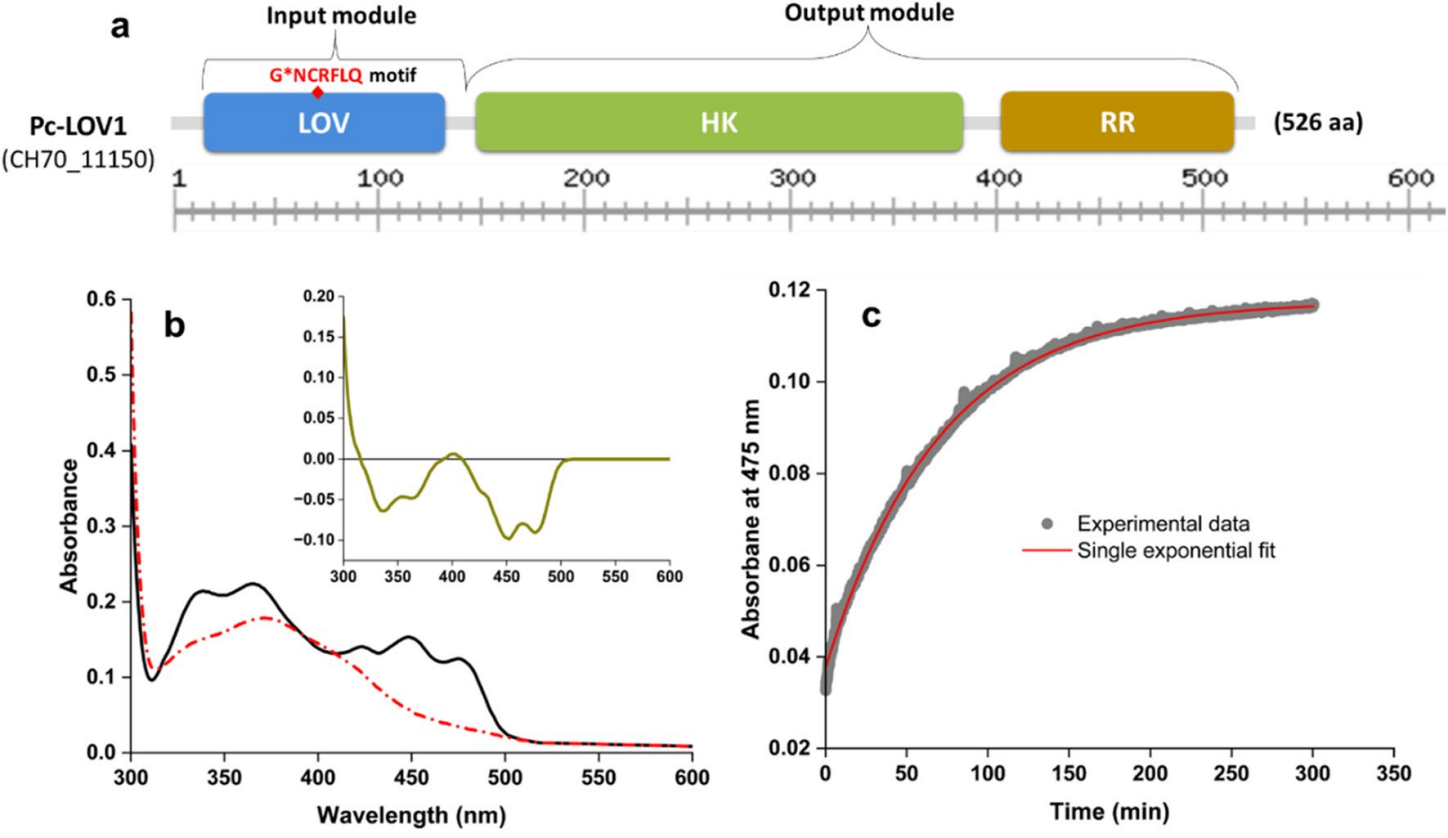
Pc-LOV1 domain architecture and photochemistry. (**a**) Domain architecture of the Pc-LOV1 protein from *P. cichorii* JBC1. The NCBI gene locus tag is included in parenthesis. Protein length, types and relative positions of all predicted Pfam domains are designated. LOV: Light, Oxygen, or Voltage; HK: histidine kinase; RR: response regulator. (**b**) Absorption spectra of the the Pc-LOV1 protein in the dark-adapted (dark state, black solid line) and blue light-illuminated (light state, red dash line). Inset shows the light–dark difference spectra of the Pc-LOV1. (**c**) The dark recovery time traces of Pc-LOV1 were monitored by measuring the absorbance at 475 nm after blue light illumination. The experimental data are represented by close circles (in gray); solid line (in red) indicates the single exponential fit of the data. All measurements were performed at 25 °C.

LOV domain-containing biological photoreceptors are found in various kingdoms. The ORF PCH70_11150 (NCBI Gene locus tag) of PcJBC1, which comprises 526 amino acids (molecular mass, 58.09 kDa), was predicted to encode an LOV-domain protein (Fig. 1a, see Supplementary Table S1 online). The domain architecture of Pc-LOV1 is similar to that of *Pseudomonas syringae* pv. *syringae* B278a (PssB278a)^9^, *P. syringae* pv. *tomato* DC3000 (PstDC3000), *X. axonopodis* pv. *citri* 306 (Xac306), and *Xanthomonas campestris* pv. *campestris* ATCC33913 (XccATCC33913)^20^ (see Supplementary Fig. S1 online). The identified Pc-LOV1 contains an N-terminal LOV domain (aa 15–132), a histidine kinase domain (HK, aa 147–383), and a C-terminal response regulator domain (RR, aa 402–515), indicating that Pc-LOV1 is a member of the histidine kinase subfamily of light-inducible two-component signal transduction systems^21,22^. The LOV domain contains the G*NCRFLQ motif (where * represents any amino acid), which contains the conserved cysteine residue involved in covalent adduct formation with an FMN chromophore^10^ (Fig. 1a, see Supplementary Fig. S1 online). The overall functional domains indicated that Pc-LOV1 is a blue-light (BL) photoreceptor. Multiple alignments of the amino acid sequence of Pc-LOV1 showed homology with LOV proteins of several plant-associated bacteria such as PssB728a (80%), PstDC3000 (80%), Xac306 (60%), XccATCC33913 (61%), McbWSM1271 (44%), RltWSM2304 (45%), and Bss168 (41%) (see Supplementary Fig. S1 and Supplementary Table S1 online).

### UV–Vis absorption spectra of Pc-LOV1

To characterize the spectral properties of Pc-LOV1, we purified the recombinant N-terminal His-tagged Pc-LOV1 protein after it was overexpressed in *E. coli* BL21. The absorption spectra of dark-state Pc-LOV1 (dark-adapted state) showed an absorption band in UV-A region with a double peak at 340 nm and 365 nm, and in the blue-region with a main absorption peak at 448 nm and two shoulder peaks at 425 and 475 nm (Fig. 1b), which are typical features of the oxidized form of the flavin within LOV proteins. The illumination of Pc-LOV1 with BL (455 nm) (light state) resulted in the loss of absorption peaks within the blue-region and the appearance of a short-wavelength absorption band around 370 nm, which indicates covalent bonding between flavin and cysteine upon BL absorption^9^. The difference in light–dark spectra indicates a canonical LOV photochemistry of Pc-LOV1 (inset of Fig. 1b). After BL illumination, the absorption peaks in the blue-region of the spectra were gradually restored during storage in the dark. The dark recovery kinetics of Pc-LOV1 were recorded by measuring the absorbance at 475 nm. The adduct state lifetimes (τ_rec_) of Pc-LOV1 determined from single exponential fits of the experimental data with the coefficient of determination R^2^ = 0.99, was 67.03 ± 4.34 min at 25 °C. (Fig. 1c).Overall, Pc-LOV1 displayed typical phototropin LOV-like UV–vis spectral properties and blue light sensitivity^23^.

### Influence of BL on the survival of PcJBC1 and its mutants

BL inactivates microbes through cell membrane damage, inactivation of virulence factors, DNA oxidation, and the changes caused by reactive oxygen species (ROS)^24–28^. Before we investigate the physiological and biological roles of Pc-LOV1 protein of PcJBC1 in response to BL, we first assessed the influence of BL intensities (1, 2.5, 5, 7.5, 10, 20, and 30 µmol/m^2^s) on the survival of wild-type (WT, PcJBC1), Pc-*lov1* deficient mutant (JBC1^Δlov1^), and complemented strain (JBC1^Δlov1^+plov1), and then determined the optimum BL treatment conditions for the subsequent experiments. The survival rates of JBC1^Δlov1^ significantly decreased when exposed to BL intensity higher than 5 µmol/m^2^s, whereas the survival of PcJBC1 and JBC1^Δlov1^+plov1 significantly decreased from 7.5 µmol/m^2^s onwards (Table 1, see Supplementary Fig. S2 online). When each strain was exposed to 10 and 20 µmol/m^2^s of BL, the survival rates of JBC1^Δlov1^ decreased to 43.5% and 7.5%, respectively, and those of PcJBC1 strain decreased to 36.1% and 3.6%, respectively. None of the strains survived when exposed to 30 µmol/m^2^s of BL. The strains were not suppressed by low intensities (1 and 2.5 µmol/m^2^s) of BL. Among the sub-lethal doses of BL, we used 2.5 µmol/m^2^s of intensity in further experiments to study the roles of Pc-LOV1 for virulence and lifestyle of PcJBC1 in response to BL.

**Table 1:** Survival rate of *P. cichorii* JBC1 cells after exposure to various intensities of blue light.

Blue light intensity (µmol/m^2^s)	Survival rate (%) of each strain*		
	PcJBC1	JBC1^Δlov1^	JBC1^Δlov1^+plov1
1.0	99.8 ± 0.25^a^	99.9 ± 0.16^a^	99.6 ± 0.76^a^
2.5	99.6 ± 0.75^a^	98.8 ± 1.20^ab^	99.7 ± 0.58^a^
5.0	99.2 ± 1.33^a^	93.8 ± 2.45^b^	97.4 ± 2.29^a^
7.5	87.0 ± 6.00^b^	87.3 ± 1.68^c^	88.4 ± 3.27^b^
10.0	36.1 ± 6.88^c^	43.5 ± 4.44^d^	39.8 ± 5.43^c^
20.0	3.6 ± 0.66^d^	7.5 ± 1.57^e^	5.5 ± 1.12^d^
30.0	0^d^	0^f^	0^d^
Dark	100^a^	100^a^	100^a^

*Overnight cultures of each strain were serially diluted and spread onto LB agar plates. The plates were incubated under various intensities (1, 2.5, 5, 7.5, 10, 20, and 30 µmol/m^2^s) of blue light and dark conditions. The number of colonies was counted 48 h after incubation at 28 °C. The data are the mean mean ± SD of triplicates and significant differences are presented by different superscript letters (a, b, c, d, e, and f) on the same column (ANOVA, *p* ≤ 0.05).

### Functions of Pc-LOV1 and BL in virulence of PcJBC1 in cabbage midrib

**Figure 2:**
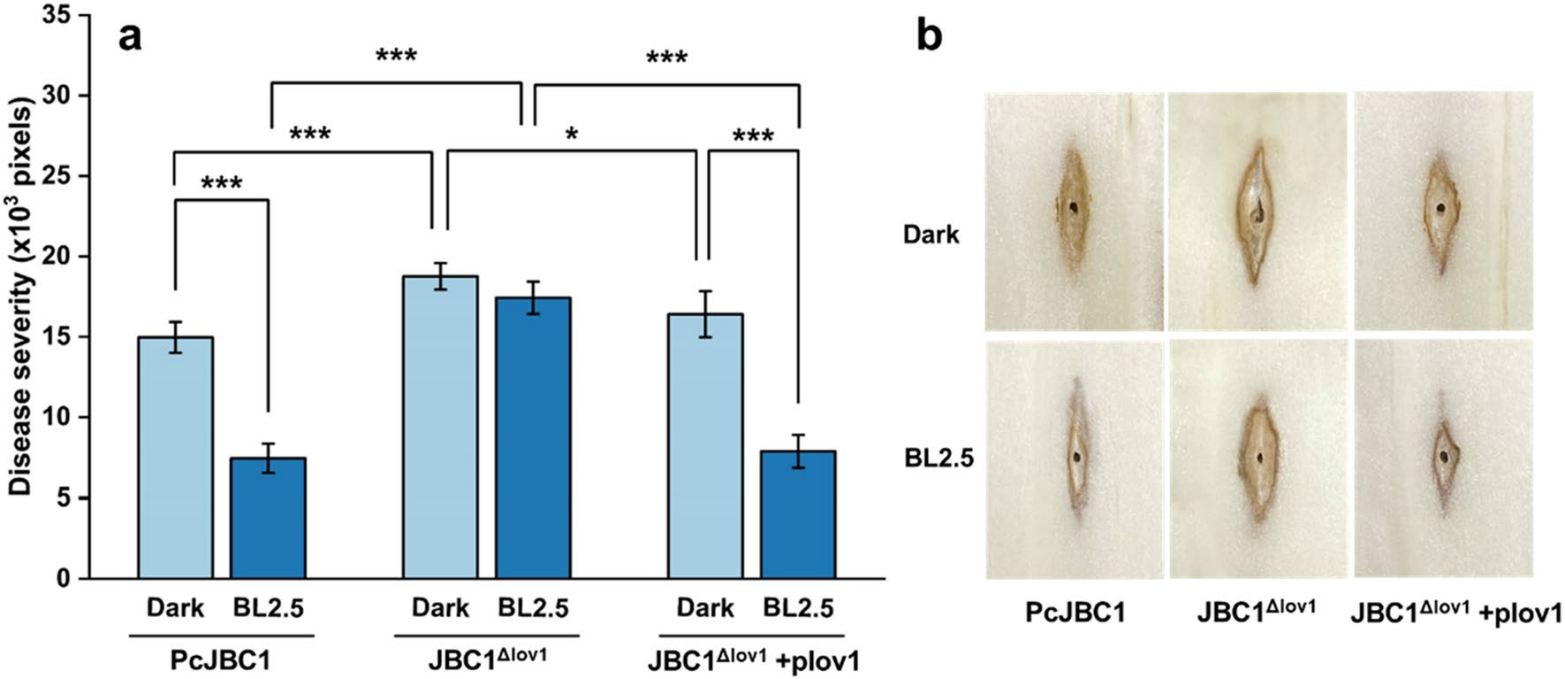
Effects of Pc-LOV1 and blue light on virulence of *P. cichorii* JBC1. (**a**) 20 μL of bacterial suspension (1 × 10^8^ CFU/mL) of PcJBC1, JBC1^Δlov1^, and JBC1^Δlov1^+plov1 was placed on the wounded cabbage midrib and incubated at 25 °C with 90% relative humidity under blue light-illuminated (2.5 µmol/m^2^s) or dark conditions. Disease severity was evaluated using Adobe Photoshop CS6. The bars presented the means ± standard deviations, and the *p*values were indicated by asterisks, *p ≤ 0.05, **p ≤ 0.01, ***p ≤ 0.001, according to Tukey’s test. (**b**) Disease symptoms appeared on cabbage midrib five days after inoculation.

To investigate the roles of Pc-LOV1 in the virulence of PcJBC1, each strain was inoculated into the cabbage midribs and incubated under BL (2.5 µmol/m^2^s) and dark conditions, and then disease incidence was assessed by measuring pixel area using Adobe Photoshop as described in Materials and Methods. Disease severity by PcJBC1 was lower under BL (7.4 × 10^3^ pixels) as compared to under dark conditions (14.9 × 10^3^ pixels). The virulence of JBC1^Δlov1^ was significantly higher compared to PcJBC1 irrespective of BL-illuminated (17.4 × 10^3^ pixels) or dark conditions (18.8 × 10^3^ pixels). The virulence of JBC1^Δlov1^+plov1 under the BL illumination was significantly suppressed (7.9 × 10^3^ pixels) compared to that under dark condition (16.4 × 10^3^ pixels) (Fig. 2). These results indicate that Pc-LOV1 negatively influences the virulence of PcJBC1 under both BL-illuminated and dark conditions.

### Influence of Pc-LOV1 and BL on the attachment to plant surfaces

**Figure 3:**
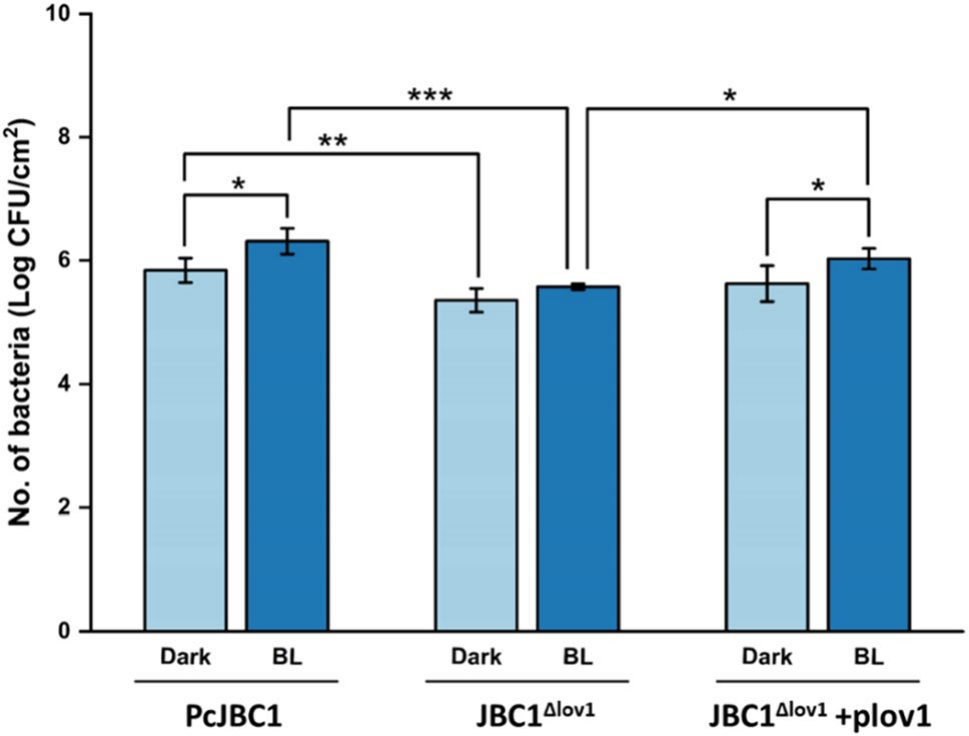
Effects of Pc-LOV1 and blue light on the epiphytic attachment of *P. cichorii* JBC1 on tomato leaves. Tomato leaves were submerged in the suspension of PcJBC1, JBC1^Δlov1^, and JBC1^Δlov1^+plov1 (1 × 10^8^ CFU/mL) for 2 h at 28 °C under blue light-illuminated (2.5 µmol/m^2^s) and dark conditions. The population of attached cells was retrieved, spread on LB agar plates, and colony numbers were counted. The bars presented the mean ± standard deviation of independent triplicates, and p values were indicated by asterisks, *p ≤ 0.05, **p ≤ 0.01, ***p ≤ 0.001, according to Tukey’s test.

Host colonization is the initial step for pathogens to enter host tissues. To determine the effects of Pc-LOV1 on the colonization of PcJBC1, the epiphytic attachment ability of each strain was assessed using tomato leaves. The adhesion of PcJBC1 was significantly increased by BL illumination (Fig. 3). The attachment of JBC1^Δlov1^ was significantly lower than those of PcJBC1 and JBC1^Δlov1^+plov1 under both dark and BL-illuminated conditions. Altogether, these results indicate that BL influences the epiphytic attachment of PcJBC1 via Pc-Lov1 regulation.

### Role of Pc-LOV1 and BL on the swarming motility

**Figure 4:**
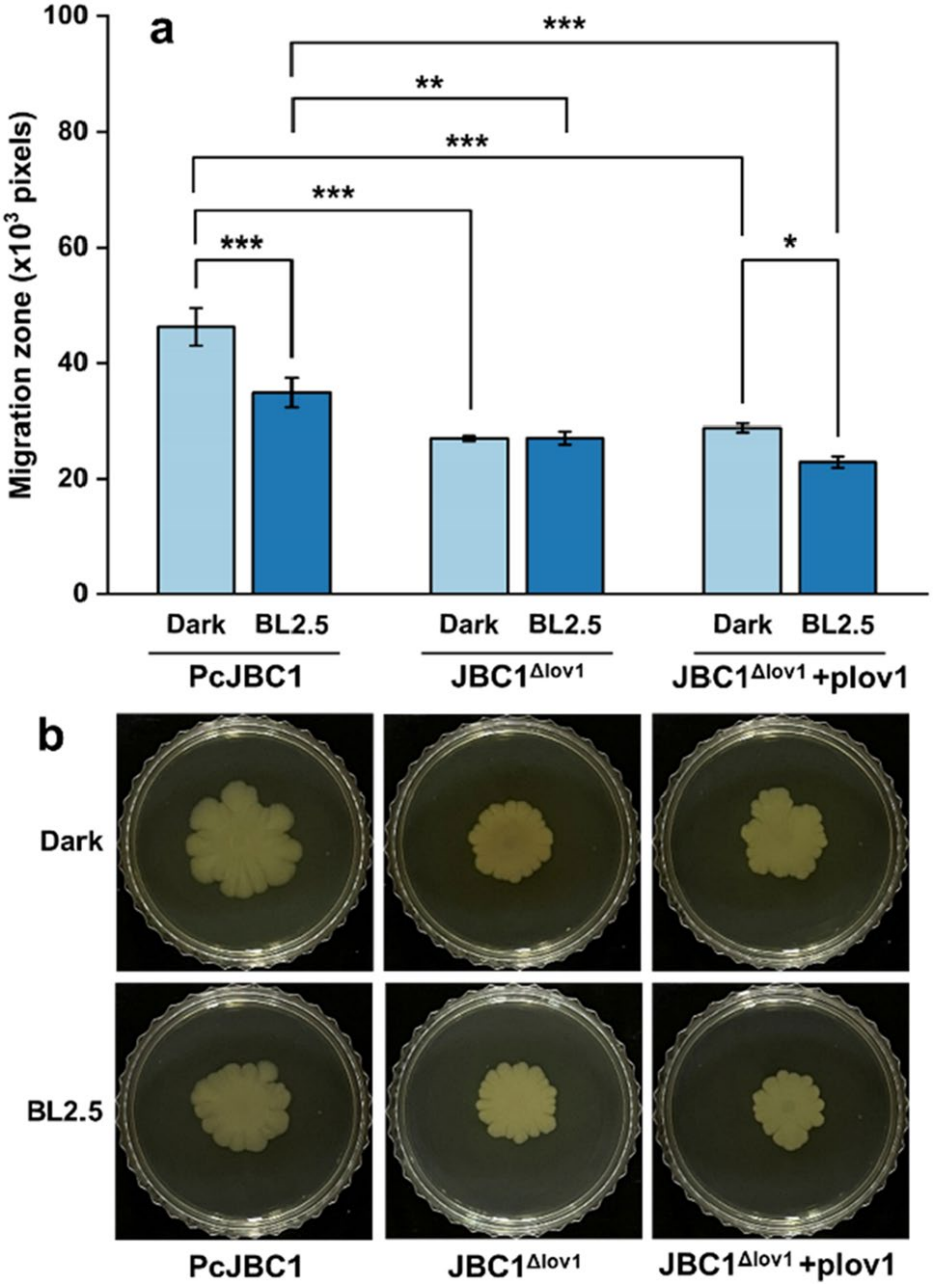
Influence of Pc-LOV1 and blue light on the swarming motility of *P. cichorii* JBC1. (**a**) Each cell suspension (10^7^ CFU/mL) of PcJBC1, JBC1^Δlov1^, and JBC1^Δlov1^+plov1 was inoculated onto LB (0.7% agar) plates, and then migration zones were measured using Adobe Photoshop cs6 magnetic lasso and histogram tools (× 1000 pixels) at 5 DAI under blue light-illuminated (2.5 µmol/m^2^s) and dark conditions at 28 °C. Data represent mean ± standard deviation of triplicate experiments and the p-values were indicated by asterisks, *p ≤ 0.05, **p ≤ 0.01, ***p ≤ 0.001, according to Tukey’s test. (**b**) The migration zones of bacterial strains in different treatments appeared five days after inoculation.

To investigate the effects of Pc-Lov1 on the swarming motility of PcJBC1, each bacterial strain was incubated on LB plates (0.7% agar) under BL-illuminated and dark conditions, and then colony size was measured by pixel area using Adobe Photoshop. Migration of PcJBC1 was more suppressed under BL illumination (34.9 × 10^3^ pixels) than under dark (46.3 × 10^3^ pixels). The migration zones of JBC1^Δlov1^ were smaller under both BL-illuminated (27.1 × 10^3^ pixels) and dark (27.0 × 10^3^ pixels) conditions compared to those of PcJBC1. The migration of JBC1^Δlov1^+plov1 was decreased by BL illumination (22.9 × 10^3^ pixels) compared to dark (28.8 × 10^3^ pixels) conditions, which is not recovered to the level of PcJBC1 (Fig. 4). These results indicate that the presence of Pc-LOV1 increases the motility of PcJBC1, and the migration zone was larger in the dark than in the BL treatment.

### Influence of Pc-LOV1 and BL on biofilm formation

**Figure 5:**
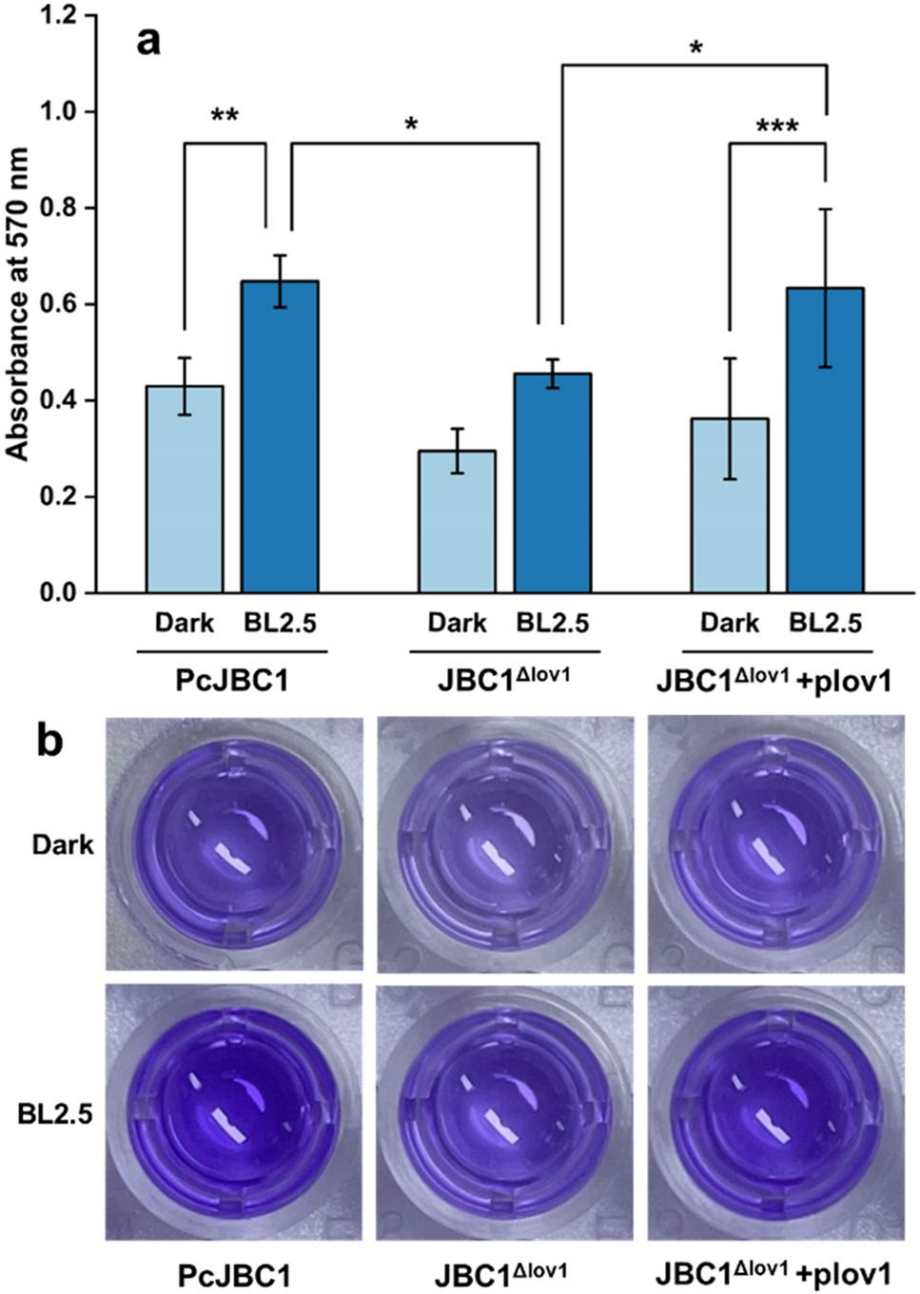
Influence of Pc-LOV1 and blue light on the biofilm formation of *P. cichorii* JBC1. (**a**) Each strain was inoculated in 96-well microtiter plates and incubated under blue light (2.5 µmol/m^2^s) and dark conditions at 28 °C. After 48 h, the biofilm was stained with crystal violet and solubilized with acetic acid followed by quantification of optical density at 570 nm using a microplate reader. The bars represented the means ± standard deviation from independent triplicate experiments, and the p-values were indicated by asterisks, *p ≤ 0.05, **p ≤ 0.01, ***p ≤ 0.001, according to Tukey’s test. (**b**) The stained biofilm in the well of the microplate from different treatments.

To investigate the role of Pc-LOV1 in biofilm formation by PcJBC1, each strain was incubated under BL and dark conditions, and the amount of biofilm formed was assessed spectrophotometrically after staining with crystal violet. BL promoted biofilm formation (OD_570_ = 0.65) in PcJBC1 compared with that in the dark (0.43) (Fig. 5). The biofilm formation by JBC1^Δlov1^ was significantly lower than that by PcJBC1 under both BL-illuminated (0.46) and dark (0.30) conditions. The biofilm formation by JBC1^Δlov1^+plov1 recovered to the amount of PcJBC1 biofilm under BL illumination (0.63). These results indicate that Pc-LOV1 positively regulates biofilm formation of PcJBC1 in response to BL.

### Effect of Pc-LOV1 and BL on exopolysaccharide (EPS) and siderophore production

The influence of Pc-LOV1 on secondary metabolites such as EPS and siderophores, which play essential roles in the virulence and nutrient absorption of PcJBC1, was assessed. EPS production by PcJBC1 under BL illumination was slightly lower than that under dark conditions (see Supplementary Fig. S3 online). JBC1^Δlov1^ produced significantly higher amounts of EPS than PcJBC1 strain under both BL-illuminated and dark conditions. Hence, Pc-LOV1 negatively influenced polysaccharide secretion by PcJBC1.

PcJBC1, JBC1^Δlov1^, and JBC1^Δlov1^+plov1 were cultured on CAS agar media under BL-illuminated and dark conditions to assess the effects of Pc-LOV1 on siderophore production. Siderophore production by PcJBC1 was more suppressed under BL illumination than under dark conditions. The JBC1^Δlov1^ produced significantly lower amount of siderophores compared to PcJCB1 under both BL-illuminated and dark conditions (see Supplementary Fig. S4 online). Therefore, the siderophore production of PcJBC1 was negatively regulated by BL and impaired by deletion of Pc-LOV1.

### Effect Pc-LOV1 on the gene expression of hypersensitive response and pathogenicity (hrp) and cichofactin

**Figure 6:**
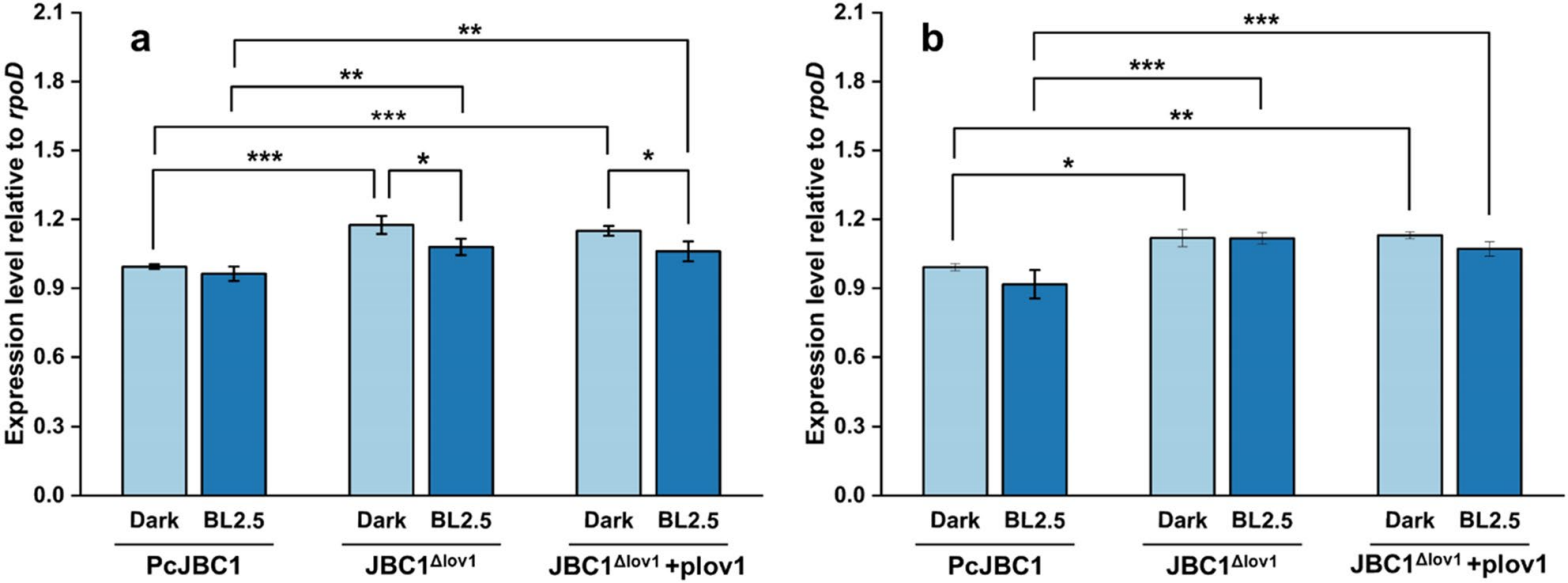
Effects of Pc-LOV1 and blue light on the the expression of genes related with hypersensitive response and pathogenicity. PcJBC1, JBC1^Δlov1^, and JBC1^Δlov1^+plov1 strains were cultured in minimal media with blue light exposure and dark conditions. After 12 h of incubation, total RNA was extracted and qPCR analysis was conducted to quantify the expression of (**a**) *hrpA* and (**b**) *hrpL*. *rpoD* was used as a reference gene. Fold differences were quantified using the ΔΔCT method with CT values normalized to the expression of each gene in PcJBC1 grown in the dark. Expression values indicate average ± SD of three independent experiments, and the p-values were indicated by asterisks, *p ≤ 0.05, **p ≤ 0.01, ***p ≤ 0.001, according to Tukey’s test.

**Figure 7:**
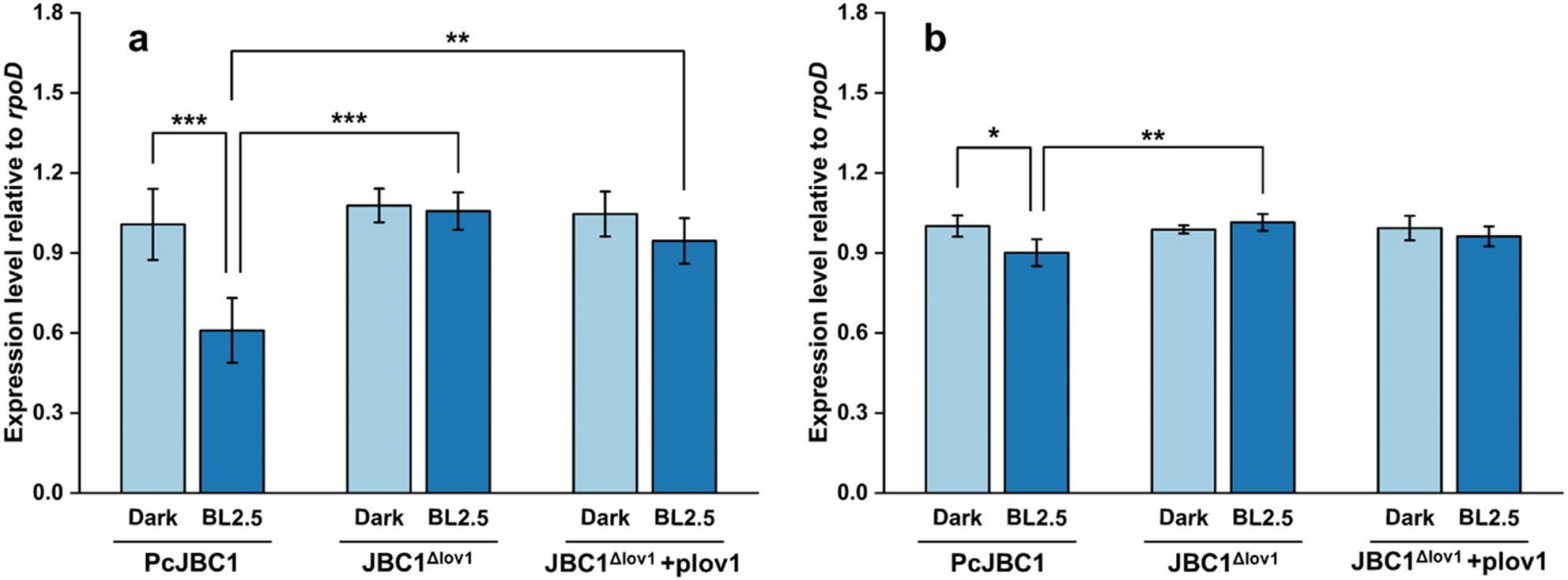
Influence of Pc-LOV1 and blue light on the the expression of genes related with cichofactin biosynthesis. PcJBC1, JBC1^Δlov1^, and JBC1^Δlov1^+plov1 strains were cultured in minimal media under blue light and dark conditions. After 12 h of incubation, total RNA was extracted and qPCR analysis was conducted to quantify the expression of (**a**) *cifA* and (**b**) *cifB*. *rpoD* was used as a reference gene. Fold differences were quantified using the ΔΔCT method with CT values normalized to the expression of genes in PcJBC1 grown in the dark. Expression values indicate average ± SD of three independent experiments, and the *p*-values were indicated by asterisks, *p ≤ 0.05, **p ≤ 0.01, ***p ≤ 0.001, according to Tukey’s test.

The wavelength and intensity of light influence the virulence of bacterial and fungal pathogens. The effects of Pc-LOV1 and BL on gene expression of the hrp pilus and regulator, and cichofactin was assessed by qRT-PCR. The expression levels of *hrpA,* a structural subunit of hrp pilus formation^29^ and *hrpL,* an alternative sigma factor that activates the transcription of the Hrp regulon^30^ in JBC1^Δlov1^ were slightly higher than PcJBC1 under both BL and dark conditions (Fig. 6). Cichofactins were involved in the pathogenicity of *P. cichorii* SF1-54^31^. The expressions of *cifA* and *cifB*, which are responsible for cichofactin biosynthesis, in PcJBC1 were more downregulated under BL illumination than under darkness, whereas their expression level in JBC1^Δlov1^ was similar between BL and dark condition (Fig. 7). Collectively, these results indicated that the virulence factors of PcJBC1 were downregulated by Pc-LOV1 under BL conditions, which was regulated by the participation and fine-tuning of several other uncharacterized light-sensing signals.

### Effect of Pc-LOV1 on gene expression for flagella formation

**Figure 8:**
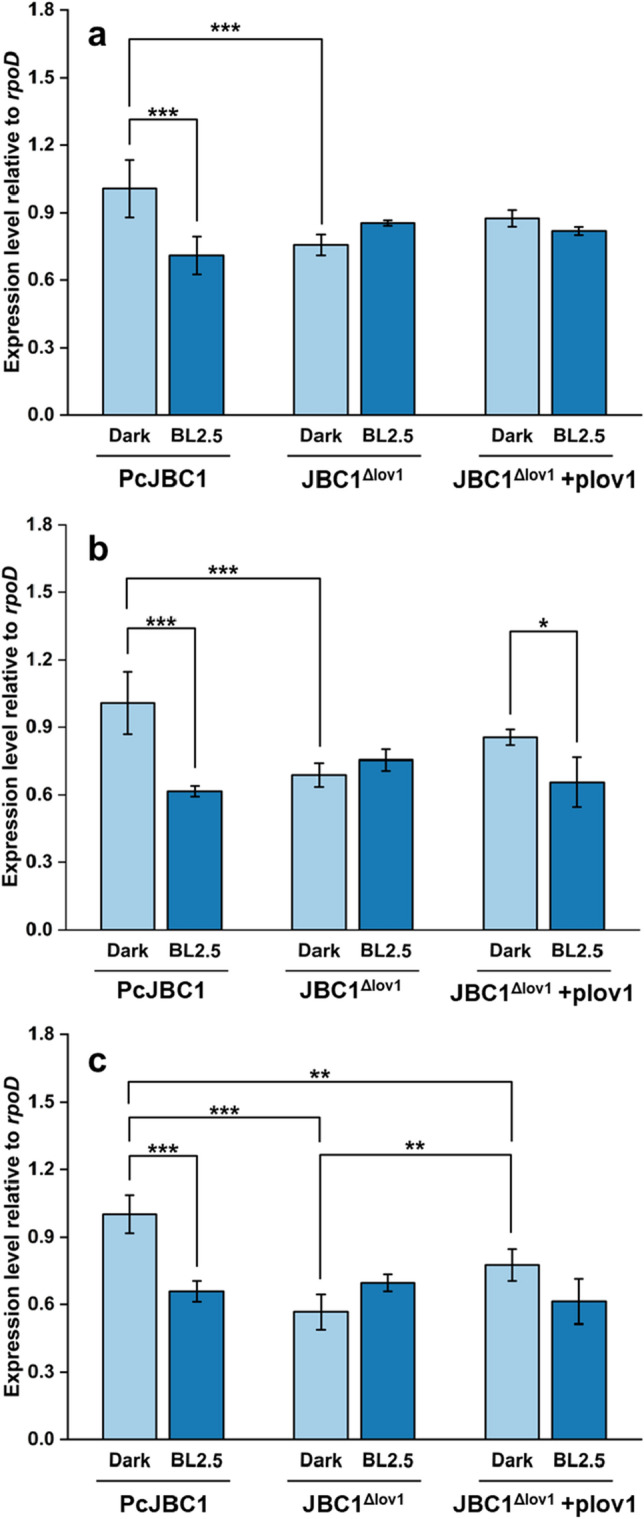
Effect of Pc-LOV1 and blue light on the expression of flagella formation-related genes. The PcJBC1, JBC1^Δlov1^, and JBC1^Δlov1^+plov1 strains were cultured in minimal media under blue light exposure and dark conditions. After 12 h of incubation, total RNA was extracted and qPCR analysis was conducted to quantify the expression of (**a**) *fliA*, (**b**) *fliG,* and (**c**) *flgJ*. *rpoD* was used as a reference gene. Fold differences were quantified using the ΔΔCT method with CT values normalized to the expression of genes in PcJBC1 grown in the dark. Expression values indicate average ± SD of three independent experiments, and the *p*-values were indicated by asterisks, *p ≤ 0.05, **p ≤ 0.01, ***p ≤ 0.001, according to Tukey’s test.

BL and Pc-LOV1 altered the swarming motility of PcJBC1. To understand the underlying modulations, we investigated the expression of genes responsible for flagella formation: *fliA,* an RNA polymerase sigma factor; *fliG,* a flagellar motor switch protein; and *flgJ,* a flagella biosynthesis chaperone. The expressions of *fliA*, *fliG,* and *flgJ* in PcJBC1 were significantly downregulated under BL illumination. The gene expressions in JBC1^Δlov1^ were significantly suppressed in comparison to those in PcJBC1 and there were no differences between BL and dark conditions (Fig. 8). These results indicate that the genes involved in flagella formation are suppressed by BL via Pc-LOV1, which corresponds to swarming motility.

### Effect of BL on *lov1* expression

Light is an important environmental signal that affects the behavior and physiology of bacterial pathogens via photoreceptors. The expression of *lov1* in PCJBC1 was similar under BL and dark conditions (see Supplementary Fig. S5 online).

## Discussion

Bacteria perceive light signals via photoreceptors and modulate many physiological and chemical processes that are essential for growth and survival^1,4^. Although many studies have reported that bacterial LOV photoreceptors are involved in many physiological processes, the roles of LOV proteins vary between the reports and the function of LOV in *P. cichorii* has remained unexplored. The domain structure and multi-alignment analyses of this study indicate that Pc-LOV1 is a member of the HKs subfamily of light-inducible, two-component signal transduction systems, which are found in *X. axonopodis*, *X. campestris*, *P. s.* pv. *syringae,* and *P. s.* pv. *tomato*^32^. LOV proteins respond to BL through BL-mediated protein–flavin adduct formation at a conserved cysteine in the LOV domain^9^. Upon sensing light signals, the input domain of the photoreceptors binds to a chromophore and initiates a change in the photoreceptor protein, which binds to DNA or proteins and triggers a signal transduction cascade. HKs autophosphorylate upon activation, facilitating the subsequent transfer of the phosphoryl group to the response regulators to induce downstream effects^7,33^.

Previous studies showed that the absorption peak at 450 nm was common to all LOV proteins in the dark state, while other absorption peaks were variable. The four LOV-HKs of *B. melitensi* and two LOV of *E. litoralis* exhibited two absorption peaks at 450 and 370 nm^9^. The recombinant LOV protein of Xac306 showed absorption maxima at 375, 450, and 475 nm in the dark^1^. The LOV of *Methylobacterium radiotolerans* showed maximum absorption at 450 nm^34^ and the aureochrom-LOV of the diatom *Phaeodactylum tricornutum* showed maximum absorption at 448, 375, and 358 nm in the dark^35^. In this study, dark-state Pc-LOV1 presented an absorbance band in the UV-A region with peaks at 340 and 365 nm and major absorption peak at 448 nm along withshoulder peaks at 425 and 475 nm in the blue-region. The absorption spectra of Pc-LOV1 corresponds to the absorption spectra of LOV proteins in *P. fluorescens* Pf01, *P. fluorescens* Pf5, *P. fluorescens* SBW25, *P. putida* W619^23^, *P. putida* KT2440^5,36^, and *Erythrobacter litoralis* (EL368-LOV-HK)^9^. BL illumination induced covalent adduct formation between the flavin chromophore and a conserved cysteine on a microsecond timescale, whereas the adduct-state decay kinetics vary from seconds to days or longer^5,9,37,38^. The adduct-state decay kinetic of each LOV protein is characterized by a dark recovery time constant called the adduct-state lifetime (τ_rec_) that depends a lot on temperature^39^. LOV proteins differ in the kinetics of adduct formation and adduct-state decay due to variations in the residues making up the binding pocket of the flavin cofactor^40–42^ and amino acids located outside the chromophore-binding pocket^43^. In our study, BL illumination of Pc-LOV1 for 1 min resulted in the loss of the absorption peaks in the visible region of the protein, and the absorption peaks was recovered after storage in the dark with τ_rec_ = 67.03 ± 4.34 min at 25 °C, indicating restoration of the oxidized state of flavin chromophores. The the adduct-state lifetime of Pc-LOV1 is faster than PpSB1-LOV (τ_rec_ ∼2400 min)^36^, but slower than YtvA (τ_rec_ = 2600 s), and is similar to YtvA-LOV (τ_rec_ = 3900 s)^44^ at the same temperature. These results indicate that Pc-LOV1 forms a typical flavin adduct in response to BL.

Plant pathogens are exposed to various wavelengths and intensities of light in the environment, including on plant surfaces. BL inactivates microbes through through many mechanisms including ROS^25–27^. The ROSs such as singlet oxygen (^1^O_2_), an oxidizing excited state of O_2_ including superoxide anion (O_2_^•−^), hydroxyl radical (HO^•^) and hydrogen peroxide (H_2_O_2_) are able to oxidize and/or oxygenate many biological molecules. Singlet oxygen is often generated by transferring electronic energy from the triplet excited state chromophores of a photosensitizer to the ground state O_2_ in biological systems^45^. However, the FMN chromophore in LOV proteins is surrounded by a protein matrix which can reduce the quantum yield (ΦΔ) by extinguishing the excited triplet state of FMN and limits oxygen diffusion towards the isoalloxazine ring of FMN^46^. In this study, we first assessed the susceptibility of PcJBC1 and Pc-LOV1-deficient mutant strains to various intensities of BL. The survival rates of PcJBC1, JBC1^Δlov1^, and JBC1^Δlov1^+plov1 significantly decreased when they were exposed to BL at intensities > 5 µmol/m^2^s. Notably, JBC1^Δlov1^ showed higher survival rates than PcJBC1 under high BL intensities of 10 and 20 µmol/m^2^s. These results indicate that the presence of Pc-LOV1 increases the susceptibility of PcJBC1 to BL, and consequently, leads to detrimental damage by BL. However, the underlying mechanisms of the increased susceptibility require further investigation.

Disease symptoms that developed in tomato and *A. thaliana* plants, as well as bacterial populations in plant tissues, were significantly reduced when bacteria were grown under BL conditions^17^. In our previous studies, PcJBC1-caused disease incidence was significantly affected by wavelength and intensity^18,19^. In this study, BL significantly reduced the PcJBC1-led disease severity in Kimchi cabbage while the JBC1^Δlov1^-led disease severity was not significantly suppressed by BL. In addition, the virulence of strains containing Pc-LOV1, i.e., PcJBC1 and JBC1^Δlov1^+plov1, was higher in darkness than under BL illumination. Kraiselburd et al.^1^ reported that the LOV-HK deficient Xac306 (Xac^∆lov^) were more virulent to orange leaves under light conditions and resulted in marked differences in symptom development compared to the WT. Río‐Álvarez et al.^17^ reported that the LOV-HK of PstDC3000 repressed type 3 secretion system (T3SS) and effectors, which probably results in reduced virulence in *A. thaliana* plants under white light (WL) and BL conditions. In this study, the virulence of Pc-LOV1-deficient mutant was significantly higher than that of the PcJBC1. Furthermore, the gene expressions of *hrpL* and *hrpA* in JBC1^Δlov1^ were higher than those in PcJBC1, which corresponded to the disease severity observed in cabbage midrib. Pauwelyn et al.^31^ reported that the *cifAB* deletion mutant, which is responsible for the biosynthesis of cichofactin, a phytotoxic lipopeptide, exhibited reduced virulence in lettuce and swarming motility, but enhanced biofilm formation compared to the WT. In our study, the expression levels of *cifA* and *cifB* in PcJBC1 decreased under BL compared to under dark condition but not in JBC1^Δlov1^, indicating the suppression of *cifA* and *cifB* expression by BL and consequent reduction in the virulence of PcJBC1. Overall, our results indicate that the presence of Pc-LOV1 and BL illumination reduce the virulence of PcJBC1, suggesting a negative role for Pc-LOV1 in the pathogenic process of PcJBC1. Disease incidence and gene expression assay results suggest that the virulence of PcJBC1 is delicately tuned by light through the participation of several other light-sensing signals and factors, which require further study.

The motility of plant pathogenic bacteria contributes to their infection potential by enabling them to reach favorable sites, such as stomata and wounds. Oberpichler et al.^47^ reported that the production of FlaA and FlaB proteins in *Agrobacterium tumefaciens* was significantly increased after light treatment. The swarming motility of Xac^∆lov^ increased irrespective of light^1^. The swarming motility of PstDC3000^∆lov^ decreased under BL but not in the dark^4^. In another study, Río‐Álvarez et al.^17^ reported that the swarming motility of PstDC3000 was truncated when grown under WL (70 μE/m^2^s) or BL (20 μE/m^2^s). The reduced motility was caused by the downregulation of gene expression involved in the synthesis of bacterial flagella and regulation of flagellar functions, which are mediated by BL via LOV^13,17^. In this study, the swarming motility of PcJBC1 was significantly reduced by BL and the motility of JBC1^Δlov1^ was suppressed under both BL-illuminated and dark conditions compared to PcJBC1. Furthermore, the expression of genes involved in flagella formation in PcJBC1 was significantly decreased by BL illumination, and Pc-LOV1 deficiency suppressed gene expression under both BL-illuminated and dark conditions. Our results indicate that gene expression for flagella synthesis is negatively regulated by BL, which supports changes in swarming motility.

Plant pathogenic bacteria must effectively colonize plant surfaces and maintain a sufficient mass of cells to cause disease in host plants. The adhesion of Xac^∆lov^ to abiotic and biotic surfaces was significantly diminished compared to that of WT, and light increased the adhesion of Xac^∆lov^ and WT compared to dark conditions, which indicates the essential role of LOV-HK for the adhesion of Xac306^1^. The biocontrol agents *Bacillus amyloliquefaciens* and *Pseudomonas chlororaphis* showed significantly higher biofilm formation when exposed to BL^48,49^. Biofilm formation and attachment of PstDC3000 to the leaves of *Arabidopsis* are favored under BL, suggesting that BL provides a signal for the bacterial switch to an attached lifestyle^17^. In this study, biofilm formation and attachment to plant surface of PcJBC1 was promoted by BL and those of JBC1^Δlov1^ were significantly decreased in comparison to PcJBC1. Collectively, our results indicate that biofilm formation and epiphytic attachment of PcJBC1 are positively regulated by Pc-LOV1 and BL. Losi and Gärtner^2^ identified BphP and Cry/PHR as well as LOV from PcJBC1 genome. We identified the protein (NCBI Gene locus tag, PCH70_12040) containing PAS domain with 38.52% sequence identity with Pc-LOV1 in the genome. In this stduty, despite the reduced attachment and biofilm formation of JBC1^Δlov1^, BL still influenced these abilities. The results suggeste the participation of other BL-sensing signals, which requires more investigation.

EPS generally contribute to the virulence of bacterial plant pathogens. The Xac^∆lov^ produced significantly high amounts of EPS compared to WT^1^. BL-upregulated genes of PstDC3000 involved in alginate biosynthesis, an EPS that increases the attachment and biofilm formation of the strain in *Arabidopsis* leaves^17^. In our study, EPS production of PcJBC1 was slightly decreased by BL, whereas JBC1^Δlov1^ produced significantly higher amounts of EPS than PcJBC1 under both BL-illuminated and dark conditions, which correspond to the results of Xac306. Negative regulation of EPS production by Pc-LOV1 may influence PcJBC1 virulence.

Bioavailable iron is an essential element for all living organisms and a critical factor in the pathogenesis of plant pathogenic bacteria. A defect in siderophore formation in *Dickeya dadantii* restricted symptom development in the inoculated leaves^50^. The virulence of pyoverdine-deficient mutants of *Pseudomonas syringae* pv. *tabaci* in tobacco plant was reduced^51^. In this study, siderophore productions by PcJBC1 and JBC1^Δlov1^ were suppressed by BL and JBC1^Δlov1^ produced significantly lower amount of siderophore compared to PcJBC1, irrespective of light. Our results indicate that siderophore production by PcJBC1 is negatively regulated by BL and suppressed by the deletion of Pc-LOV1, although it is still influenced by BL signals. Changes in EPS and siderophore production suggest that BL influences the secretion or production of metabolites in PcJBC1 via Pc-LOV1. Notably, in our swarming motility assays, the colors of the colonies differed with or without BL-illumination and Pc-LOV1. These results indicate that pigment production is also influenced by Pc-LOV1, which requires further investigation.

Bonomi et al.^15^ reported that LOV protein levels in *R. leguminosarum* remain unchanged under light and dark conditions. In the present study, PcJBC1 showed the same level of *lov1* expression with or without BL, which indicating that BL does not induce the expression of *lov1*.

In this study, we investigated the roles of Pc-LOV1 and BL in virulence and the ecological behavior and physiology associated with the virulence of PcJBC1. Pc-LOV1 and BL negatively influenced the virulence, virulence-related gene expression such as *cifA*, *cifB*, *hrpA*, and *hrpL*, swarming motility, flagella formation-related gene expression such as *fliA*, *fliG*, and *flgJ,* EPS production, and siderophore biosynthesis of PcJBC1. At the same time, the traits relevant to colonization on plant surface, such as adhesion to the plant tissue and biofilm formation were positively regulated. Bacterial pathogenicity is a result of a combination of many factors^52^. How the contradicting traits influence on the overall virulence of PcJBC1 requires further studies. The variable responses of these features to BL indicate that other photosensory proteins possibly participate in light sensing and downstream signaling. The signaling pathways and networks with other candidate photoreceptors, and roles of Pc-LOV1 in the dark remain to be explored.

## Materials and methods

### Identification of LOV1 sequence and domain analysis

The open reading frame (ORF) (NCBI Gene locus tag, PCH70_11150) in the PcJBC1 genome (NCBI Acc. No. CP007039.1)^53^ was identified as a putative LOV1 photoreceptor protein (Pc-LOV1) using CDD analysis at NCBI. The amino acid sequence and domain architecture of Pc-LOV1 was compared with *P. s.yringae* pv. *syringae* B278a (PssB278a) (NCBI Acc. No ASM1839437v1), *P. syringae* pv. *tomato* DC3000 (PstDC3000) (AE016853.1), *X. axonopodis* pv. *citri* 306 (Xac306) (ASM96121v1), *X. campestris* pv. *campestris* ATCC33913 (XccATCC33913) (ASM1338837v1), *Mesorhizobium ciceri* bv. *biserrulae* WSM1271 (McbWSM1271) (ASM161884v1), *Rhizobium leguminosarum* bv. *trifolii* WSM2304 (RltWSM2304) (ASM430655v1), and *Bacillus subtilis* subsp. s*ubtilis* 168 (Bss168) (ASM904v1), which were retrieved from NCBI. All sequences were analyzed for the presence or absence of domains using the Pfam server (http://pfam.xfam.org/). The deduced amino acid sequences were aligned and compared using Clustal Omega program^54^.

### Deletion of *lov1* using CRISPR-CAS9

*Lov1* (from start to stop codons) of PcJBC1 was knocked out using the pCasPA/pACRISPR system developed by Chen et al.^55^ with minor modifications^56^. The gRNA sequence was replaced with primers sgLOV1-F and sgLOV1-R (see Supplementary Table S2 online) through Golden Gate Assembly and used for the transformation of *Escherichia coli* TOP10 using standard techniques. The transformants with the pACRISPR-sgRNA-*lov1* plasmid were screened on ampicillin plates (150 μg/mL) and confirmed through PCR using primers sgLOV1-F/R-Amp. Simultaneously, the upstream and downstream regions of *lov1* (500 bp) were individually amplified by PCR. Equal amounts of digested pACRISPR-sgRNA-*lov1* with *Xba*I and *Xho*I were assembled with upstream and downstream fragments using Gibson assembly and cloned into *E. coli* TOP10. The construct was confirmed by PCR using the LOV1-Us-F/LOV1-Ds-R primer set and by sequencing. The pACRISPR-sgRNA-*lov1*-Us-Ds plasmid was electroporated into the PcJBC1-pCasPA cells (see Supplementary Table S3 online). The electroporated cells were recovered by adding 1 mL LB broth, incubated at 30 °C for 1–2 h, and then plated on LB agar plates containing 100 μg/mL tetracycline and 150 μg/mL ampicillin. The *lov1*-defective mutant (JBC1^Δlov1^) was verified for correct deletion through PCR and sequencing with the LOV1-Us-F/LOV1-Ds-R primers (see Supplementary Table S2 online).

### Complementation of knock-out mutant

The photoreceptor mutant (JBC1^Δlov1^) was complemented by amplifying *lov1* along with its promoter region from genomic DNA of PcJBC1 with primer sets, LOV1-HindIII-500Us-F and LOV1-BamHI-R (see Supplementary Table S2 online). The PCR reaction was conducted as follows; 5 min for initial denaturation at 95 °C; 30 cycles of 40 s of denaturation at 95 °C, 40 s for annealing at 60 °C, and 3 min of extension at 72 °C and final extension at 72 °C for 10 min. The PCR product was purified using an Expin Gell SV kit (GeneAll Biotechnology Co., Seoul, South Korea) and cloned into the pUCP18 vector using *Hind*III and *Bam*HI restriction sites. *lov1* was cloned in a reverse orientation to the *lacZ* promoter, securing gene expression from the native promoter rather than from the *lacZ* promoter^56^. The hybrid plasmid pUCP-*lov1* was transferred into the mutant strain (JBC1^Δlov1^) by electroporation yielding a complemented strain (JBC1^Δlov1^+plov1).

### Creation of construct for Pc-LOV1 overexpression

To express the Pc-LOV1 recombinant protein, *lov1* was amplified from PcJBC1 genomic DNA using PCR with the primers BamHI-Lov1-1F and HindIII-Lov1-1581R (see Supplementary Table S2 online) and cloned into the pET-28a plasmid. The plasmids were then transformed into electrocompetent *E. coli* TOP10*.* The transformants with the pET28a_*lov1* plasmid were screened on kanamycin plates (50 μg/mL) and confirmed through PCR and sequencing using primers T7-promoter-F/T7-terminator-R. The isolated pET28a_*lov1* plasmid was transformed into competent *E. coli* BL21 cells. The recombinant *E. coli* BL21 cell was confirmed through PCR and grown overnight (approximately 16 h) in LB with kanamycin (50 μg/mL) at 37 °C. The overnight culture (2%, *v*/*v*) was seeded into 300 mL of fresh LB medium with kanamycin, and riboflavin was added directly to the media at the final concentration of 3 µM. The bacteria were grown to OD_600_ of 0.5–0.8 at 37 °C and isopropyl β-D-1-thiogalactopyranoside was supplemented to a final concentration of 10 µM. The recombinant Pc-LOV1 protein of PcJBC1 Pc-LOV1 was expressed in *E. coli* BL21 cells at 18 °C for 22 h in the dark.

### Isolation and purification of Pc-LOV1

Pc-LOV1 was purified according to the method described by Goett-Zink et al.^57^ with minor modifications. Briefly, the Pc-LOV1-overexpressing cells were harvested via centrifugation at 4,000 RPM for 10 min at 4 °C. Cell pellets were resuspended in 350 mM phosphate buffer (pH 8.0) containing 300 mM NaCl, 20 mM imidazole, 20% glycerol, and protease inhibitor (PMSF, phenylmethylsulfonyl fluoride; Sigma-Aldrich), and lysed using a sonicator with every cycle for 4 min consisting of repeating 2 s pulses at 30% power and 2 s pause, three cycles in total. The supernatant obtained following a 15-min centrifugation at 15,000 RPM and 4 °C was incubated with 5 mM FMN on ice for 20 min^58^. The mixture was mixed with binding buffer (4:1, v/v) and loaded into a HisTrap FF column (GE Healthcare, USA) using a pump. The column was rinsed with 30 column volumes of binding buffer (50 mM phosphate buffer, 20 mM imidazole, 300 mM NaCl, and 20% glycerol; pH 8.0). The recombinant protein Pc-LOV1 was recovered from the column with a gradient between the binding buffer and elution buffer (50 mM phosphate buffer, 300 mM NaCl, 250 mM imidazole, and 20% glycerol; pH 8.0). The purity of the eluted fractions was observed by sodium dodecyl sulfate–polyacrylamide gel electrophoresis. Pure fractions were pooled and the elution buffer was exchanged to 10 mM phosphate buffer, pH 8.0supplemented 1 mM dithioerythritol (DTT), 300 mM NaCl, and 20% glycerol using a Pierce™ Protein Concentrator (10 K MWCO PES, Thermo Scientific, USA). The protein concentration was determined using the Bradford method^59^.

### UV–Vis spectrophotometry analysis

The absorbance spectra of purified Pc-LOV1 were recorded using a UV–Vis Spectrophotometer (Agilent, USA). The assay was recorded in the dark and after 1 min of illumination with BL (455 nm, 120 µmol/m^2^s). Dark recovery kinetics was measured from the BL-illuminated protein by monitoring the absorbance at 475 nm as a function of time^5,36^ at 25 °C for 5 h. Dark recovery time traces were fitted into a single exponential function that gave the dark recovery lifetime (τ_rec_) using following Eq. (5); Abs = A_0_+A_1_e(^−t/τ^_rec_), where Abs- measured absorbance at 475 nm, t is the time since illumination, τ_rec_ is the time constant referred to as the adduct lifetime by employing OriginPro 2024 (OriginLab Corp., Northampton, Massachusetts, USA)^23^. The measurements were performed in triplicate using three separate protein preparations.

### Exposure of BL to bacterial cells

A light-emitting diode (LED) that emits BL (wavelength range: 448–475 nm, with typical light emission at 458 nm) was used in this study^18,49^. The LED light-sources were connected to a circuit box enabling the regulation of the light intensity, and photosynthetic photon flux density (PPFD), which quantifies photons as µmol/m^2^s, was measured using a quantum sensor (LI-190 SB; Li-Cor, Lincoln, Nebraska, USA) as necessary. The strains PcJBC1, JBC1^Δlov1^, and JBC1^Δlov1^+plov1 were cultured in LB broth with vancomycin (100 mg/L) for 24 h at 28 °C. Bacterial cells were collected via centrifugation at 4,000 RPM for 5 min, resuspended in sterilized distilled water, and adjusted to 0.2 OD_600_ (1 × 10^8^ CFU/mL). Serially diluted (tenfold) suspensions were spread onto LB agar plates. The plates were incubated under BL with different intensities (1, 2.5, 5, 7.5, 10, 20, and 30 µmol/m^2^s) and dark conditions. The colonies were counted after a 48-h incubation at 28 °C. The experiment was performed in triplicates.

### Virulence assay using midrib of cabbage

Each strain (PcJBC1, JBC1^Δlov1^, and JBC1^Δlov1^+plov1) was cultured in LB broth with vancomycin and bacterial cells were harvested via low-speed centrifugation and diluted into OD_600_ = 0.2 (1 × 10^8^ CFU/mL) in sterile 10 mM MgCl_2_ buffer^18^. Each bacterial strain was inoculated into the midribs of cabbage as described by Huong et al.^56^. Briefly, detached midribs of Kimchi cabbage (*Brassica rapa* subsp. *pekinensis*) obtained from the local market were surface-disinfected with 70% ethanol, washed twice with sterile distilled water (DW), and blot-dried with tissue paper. A toothpick was used to induce a 0.5**-**mm deep wound on the surface-disinfected midribs, and a 20 µL cell suspension of each strain was applied to the wound. The inoculated midribs were allowed to air-dry for 10–15 min and placed on the lid of Petri dishes, which were laid on the two layers of paper towels moistened with sterile DW in the plastic boxes with dimensions of 30 × 20 × 10 cm^3^, and incubated at 25 °C and > 90% relative humidity under BL (2.5 µmol/m^2^s) and in darkness. Diseased areas and symptoms were evaluated five days after incubation (DAI) by measuring pixel areas using the Magnetic Lasso and histogram tools of Adobe Photoshop CS6^60^. Five wounds were recorded for each treatment, with three replicates per treatment. All experiments involving plants were carried out following relevant guidelines.

### Leaf attachment assay

A leaf attachment assay was performed following the method described by Kroupitski et al.^61^ with minor modifications. Briefly, overnight cultures of each strain were resuspended in sterile DW at a concentration of 1 × 10^8^ CFU/mL. Whole leaves of tomato were submerged in the bacterial suspension for 2 h at 28 °C under BL-illuminated (2.5 µmol/m^2^s) and dark conditions, and rinsed twice with sterile DW to remove any unattached bacteria. Six random leaf disks (8 mm in diameter) were cut and macerated with a sterilized mortar and pestle in 0.9% NaCl solution to release the attached bacteria from the leaves. The serially diluted suspensions were spread onto the LB agar plates containing vancomycin (100 mg/L) for PcJBC1 and JBC1^∆lov1^, and vancomycin and ampicillin (100 mg/L) for JBC1^∆lov1^+plov1 strain. After 48 h of incubation at 28 °C, the number of colonies was quantified and presented on a log10 scale (CFU/cm^2^). The experiment was conducted in triplicates.

### Swarming assay

The swarming motility assay was performed according to the method described by Nagendran and Lee^62^ with minor modifications. Briefly, the overnight cultures of PcJBC1, JBC1^Δlov1^, and JBC1^Δlov1^+plov1 were sub-cultured with 2% inoculum in fresh LB broth and grown till late exponential phase. Bacterial cells were collected via low-speed centrifugation and resuspended in sterile DW at a final concentration of 1 × 10^7^ CFU/mL. A 3 μL aliquot was inoculated onto the center of LB agar (0.7% agar, *w*/*v*) plates and incubated under BL-illuminated and dark conditions at 28 °C. The migration zones were recorded at 5 DAI by measuring the pixel areas using the Magnetic Lasso and histogram tools of Adobe Photoshop CS6^60^. All experiments were performed in triplicates.

### Biofilm formation assay

Static biofilm formation was assessed following the method described by Merritt et al.^63^ with small modifications. Each strain was grown to 0.2 OD_600_ and diluted 100-fold with fresh LB broth supplemented with 1 mM MgSO_4_. One hundred μL of each diluted culture was distributed into 96-well microplates and incubated for 48 h at 28 °C without shaking under BL (2.5 µmol/m^2^s) and darkness. After incubation, the bacterial cells were decanted and eliminated by washing with phosphate-buffered saline. Subsequently, the wells were stained with 125 μL of 0.1% w/v crystal violet. After 10 min of incubation at room temperature, the microplates were rinsed thrice with DW and air-dried to eliminate excess water. The stained biofilm mass was dissolved by adding 200 μL of 30% acetic acid and incubating for 15 min at room temperature. A 125 μL of each dissolved mixture was transferred to a fresh microplate and value of optical density at 570 nm was measured using a microplate reader. The experiment was conducted in triplicates.

### EPS production assay

EPS production was quantified as described by Zimaro et al.^64^ with minor modifications. Briefly, the bacterial strains were cultured in 100 mL of LB broth in 250-mL flasks at 180 RPM and 28 °C for 72 h under BL-illuminated (2.5 µmol/m^2^s) and dark conditions. The bacterial mass was centrifuged at 10,000 RPM for 15 min and the supernatant was collected. The cell-free supernatant was supplemented with 1% (*w*/*v*) of KCl and three volumes of 95% ethanol (*v*/*v*) and left overnight at 4 °C. The precipitated crude EPS was collected via centrifugation at 10,000 RPM for 15 min, then the pellet was dried overnight at 37 °C and weighed. EPS quantities were expressed in milligrams per liter of culture. The experiments were independently repeated thrice.

### Siderophore production assay

Siderophore production by each strain was determined using the modified chrome azurol S (CAS) agar method^65^. Briefly, the CAS solution mixed with 1 mM ﻿FeCl_3_ solution was added to the hexadecyltrimethylammonium bromide (HDTMA) solution and autoclaved. The mixture (100 mL) was added to 900 mL autoclaved LB agar medium at pH 6.8. Overnight grown cultures (20 µL) of PcJBC1, JBC1^Δlov1^, and JBC1^Δlov1^+plov1 in LB broth were spot-inoculated onto the sterile paper disks laid on CAS plates, and the plates were incubated at 28 °C for three days under BL-illuminated (2.5 µmol/m^2^s) and dark conditions. The diameter of the orange halo zone formed around the paper disks was used to evaluate siderophore production by the bacterial strains. All experiments were performed in triplicates.

### RNA isolation and qPCR for gene expression analysis

Gene expression analysis was conducted following the method described by Rajalingam and Lee^19^ with minor modifications. Briefly, PcJBC1, JBC1^Δlov1^, and JBC1^Δlov1^+plov1 were cultured overnight in 10 mL LB medium at 28 °C in a shaking incubator and collected via centrifugation at 4,000 RPM for 10 min. Bacterial cells were washed once in minimal medium (MM)^26^ and resuspended to 0.6 at OD_600_ in 10 mL MM supplemented with fructose in 50-mL flasks. Each flask was exposed to BL (2.5 µmol/m^2^s) or wrapped with aluminum foil to secure darkness^19^. After incubation for 12 h at 180 RPM in a shaking incubator at 28 °C, the bacterial cells were collected, and the RNA was extracted from each sample using TRI reagent solution kit (Ambion, USA). The quality and quantity of extracted RNA samples were analyzed using an electropherogram (Agilent Bioanalyzer 2100). The expression levels of genes encoding putative photoreceptor (*lov1*), hrp pilus and regulator (*hrpL* and *hrpA*), phytotoxic lipopeptides (*cifA, cifB*), and flagella formation (*fliA*, *fliG*, and *fliJ*) were analyzed by qPCR using primers for each gene (see Supplementary Table S2 online).

### Statistical analysis

All the experiments were performed using a completely randomized design. Statistical differences among the means of the experimental treatments were analyzed by one-way analysis of variance (ANOVA) followed by Tukey’s multiple comparison test at the *p* ≤ 0.05 level of significance using Minitab version 16.2.0 software^66^.

### Supplementary Information

Supplementary Information.

## Data Availability

The datasets used and/or analyzed in the current study are available from the corresponding author upon reasonable request.
