# Supplementary material for: LOV1 protein of Pseudomonas cichorii JBC1 modulates its virulence and lifestyles in response to blue light
Source: Sci Rep. 2024 Jul 8;14:15672. doi: 10.1038/s41598-024-66422-1 (PMC11231323; doi:10.1038/s41598-024-66422-1)
Supplement: Supplementary file 1 — Supplementary Information. [file 41598_2024_66422_MOESM1_ESM.pdf]

Supplementary Information

LOV1 protein of *Pseudomonas cichorii* JBC1 modulates its virulence and lifestyles in response to blue light

Nguyen Van Khanh[1], Yong Hoon Lee[1, 2,*]

```
PcJBC1          ---------------MNNRVTGDISHPQKDIFFAAVETTRMPMIFTDPHRPDNPIIFANQ    45
PssB728a        MS------EN--KTRVDNAATGDIKHQGKDIFFAAVETTRMPMIVTDPNRPDNPIIFANR    52
PstDC3000       MS------EN--KTRVDNAATGDIQHQGKDIFFAAVETTRMPMIVTDPNRPDNPIIFSNR    52
Xac306          MNDPGHGELR--APHISESRSLPVEKHRSDIFFAAVETTRMPMTVTDPHLPDNPIVFANR    58
XccATCC33913    MNDSGYGEPR--APLIGESQTLPVSKHRSDIFFAAVETTRMPMTVTDPYLPDNPIVFANR    58
McbWSM1271      ME---WKRTREPADAWHMTDDLHVEHGKGDPFAAAIRATRMSMIITDPRQQDNPIVFAND    57
RltWSM2304      MTTHSTKKLHG---DLPSASSKTALADRKELAAVAFERTRMPMVVTDARKPDLPIVLANK    57
Bss168          ---------------MASFQSF-GIPGQLEVIKKALDHVRVGVVITDPALEDNPIVYVNQ    44
                                             :    *.  .*: : .**    * **:  *

PcJBC1          AFLEMTGYSADEIVGRNCRFLQGPETDRAVVALRQAIADKQDIATEILNYKKDGSSFWN    105
PssB728a        AFLEMTGYASEEIIGSNCRFLQGPDTDRTAVQSIRDAIDQRVDISTEILNYRKDGSSFWN    112
PstDC3000       AFLEMTGYTAEEILGTNCRFLQGPDTDPAVVQSIRDAIAQRNDISAEIINYRKDGSSFWN    112
Xac306          AFLEMTGYAADEVIGNNCRFLQGPETDPASISDVRESIESRREFATEVLNYRKDGSSFWN    118
XccATCC33913    AFLEMTGYAADEIIGNNCRFLQGPETDRQAVADVREAIDNRREFATEVLNYRKDGSTFWN    118
McbWSM1271      AFLRLTGYERHEVLGRNCRFLQGPKTDKAAVEQIRAAIEDETDVSVDILNYRKDGSTFWN    117
RltWSM2304      SFLDLTGYAADEVVGRNCRFLQRPATSPIAVAEIRASIAEERDVSVEILNYKKSGEPFWN    117
Bss168          GFVQMTGYETEEILGKNCRFLQGKHTDPAEVDNIRTALQNKEPVTVQIQNYKKDGTMFWN    104
                .*: :***  .*::* ******   *.   :  :* :: ..  .:.:: **:*.*  ***

PcJBC1          ALFMSPVFNDNGDLVYFFASQLDVSRRRDAEDALRQAQ---------------KMEALG    149
PssB728a        ALFISPVYNDAGELIYFFASQLDISRRRDAEEALRQAQ---------------KMEALG    156
PstDC3000       ALFISPVYNDAGDLIYFFASQLDISRRKDAEEALRQAQ---------------KMEALG    156
Xac306          ALFISPVFDDKGNLVYFFGSQLDVSRRRDAEDALRQAQ---------------KMEALG    162
XccATCC33913    ALFVSPVFDDTGKLVYFFGSQLDVSRRRDAEDALRQAQ---------------KMEALG    162
McbWSM1271      ALYISPVSNDKGDLQFFFASQLDVSDRKQSESRINAEKDRFEKAVKERTAELEAALEAQT    177
RltWSM2304      RLHLSPIHGDDGRILYFFGSQIDMTEYRRVEALEAS---------------------EH    155
Bss168          ELNIDPMEIE--DKTYFVGIQNDITKQKEYEKLLEDSLTEITALS-TPIVPIRNGISALP    161
                 * :.*:  :     :*.. * *::  :  *

PcJBC1          QLTGGIAHDFNNLLQVMGGYIGLIDSATDKSSIDVPRIKRSVEHAKSAVDRASTLTKQLL    209
PssB728a        QLTGGIAHDFNNLLQVMGGYIDLIGSAAEKPVIDVQRVQRSVHHAKSAVERASTLTKQLL    216
PstDC3000       QLTGGIAHDFNNLLQVMGGYIDLIGSAAEKPVIDVQRVQRSVYHAKSAVERASTLTKQLL    216
Xac306          QLTGGIAHDFNNLLQVMSGHLEVIQTMASAGGSSAERIAFSAEHAAAAAAKAATLTQQLL    222
XccATCC33913    QLTGGIAHDFNNLLQVMSGHLEFIQMMVSNGNGSPDRIAVSAEHAAAAAAKAATLTQQLL    222
McbWSM1271      TLLHEVDHRVKNNLQMISSLIIMQRRT-----IRDEAIRRSLTTMLERIEALSTVHRRLY    232
RltWSM2304      RLLMEVDHRSKNVLAIVDSIVRLSNADD--AALYAAA----IQHRVQALARAHN----LL    205
Bss168          LVGNLTEERFNSIVCTLTNILSTSKDD--YLIIDLSGLAQVNEQ---------TADQIF    209
                 :     .  :. :  : . :                                     :

PcJBC1          AFARKQKLQGRVLNL-NQVVTGMEALIERTFGAEVPVEYSLEPALLNCRLD-PSQAEVAL    267
PssB728a        AFARKQKLQGRVLNL-NGLVSTTEPLIERTFGPEVIIETDLDPALKNCRID-PTQAEVAL    274
PstDC3000       AFARKQKLQGRVLNL-NGLVSIVEPLIERTFGPEVAIETDLEPALKNCRID-PTQAEVAL    274
Xac306          AFSRKQKLRGRVVNL-NGLVAGMTNMAERALGGGVTLRQSLEEGLWNCQID-TTQAEVAL    280
XccATCC33913    AFSRKQKLRGRVVNL-NGLVSGMNNMAERALGGGITLRQALEERLWNCQID-TTQAEVAL    280
McbWSM1271      QSKDVSKFD--VADFAKDLVT---DLLTASGRSEISPALDLEPIVISAEKA-TP---VAL    283
RltWSM2304      AERGWSNIS--VEELVRLQVTPFAATRVALNGPDIRLP------------A-TVVQPVAL    250
Bss168          KLSHLLKLTGTELI-----ITG--------IKPELAMKMNKLDANFSSLKTYSNVKDAVK    256
                      ::           ::             :                      ..

PcJBC1          -LNILI-NARDALIGRQDRRVFIETRNIIVKDLASVSYD--GLLPGSYVSISITDNGIGM    323
PssB728a        -LNIFI-NARDALIGRLNPKIFIETRNLVVDELANMSYD--GLLPGRYVSIAVTDNGIGM    330
PstDC3000       -LNIFI-NARDALIGRENPKVFIETRNLLVDELANMSYD--GLLPGRYVSIAVTDNGIGM    330
Xac306          -LNVLI-NARDAMAQAERKEVTVQTQNVEITGHDLAMYH--QLAPGRYVSIAVTDTGSGM    336
XccATCC33913    -LNVLI-NARDAMANAERKEVMVQTQNVEITSHDLSMYH--QLAPGRYVSIAVTDSGSGM    336
McbWSM1271      MVNELVTNAL--------KHAFKSKPDGM--SAGRLGIK--MSQPDGHLNIEVSDDGVGM    331
RltWSM2304      VLHELAVNAAR--------HGA------LAKPLGKLSIDWAPGQSDDGFRLRWKEVGSGP    296
Bss168          VLPIM-------------------------------------------------------    261
                 :  :
```

```
PcJBC1          PASIRDRVMDPFFTTKEEGQGTGLGLSMVYGFAK-QS-GGAARIYSEEDVGTTLRLYFPV    381
PssB728a        PASIRDRVMDPFFTTKEEGKGSGLGLSMVYGFAK-QS-GGAARIYTEEGVGTTLRLYFPV    388
PstDC3000       PASIRDRVMDPFFTTKEEGKGSGLGLSMVYGFAK-QS-GGAARIYTEEGVGTTLRLYFPV    388
Xac306          PPEVVSRVMEPFFTTKEEGQGTGLGLSMVYGFVK-QS-GGTVRIYSEVGEGSTVRLYFPA    394
XccATCC33913    PPEVVSRVMEPFFTTKEEGQGTGLGLSMVYGFVK-QS-GGTVRIYSEVGEGTTVRLYFPA    394
McbWSM1271      ADANGDA---------------SFGMRLIKSLAR-QL-HA-EIEWRDTGPGTKVVISIPN    373
RltWSM2304      PPKSTKR---------------GFGTVIVGAMVEKQLKGQFEKTWLDDGL--LIEIEVPA    339
Bss168          ------------------------------------------------------------    261

PcJBC1          DDASISIHDHATPVASK-TGSERILIVEDRPDVAELAKMVLEDYGYVTDMVLNAREALKR    440
PssB728a        DEAVLSKNDPPKASERRIGSSERILIVEDRPDVAELAKMVLDDYGYVSEIVLNAREALKR    448
PstDC3000       DEAGLTNTESPQASDRRLGSSERILIVEDRPDVAELAKMVLDDYGYVSEIVLNAREALKK    448
Xac306          SSEFENDLQIAKSRAIDKGGNETILVVEDKQDVAVVARMFLENAGYRILSASSGREAMEI    454
XccATCC33913    SSDYENALPSTKNRALDKGGNETILIVEDKEDVAVVAKMFLEGAGYRALSASSGREAIEV    454
McbWSM1271      EPQEKGNVS---------------------------------------------------    382
RltWSM2304      TGSRPV------------------------------------------------------    345
Bss168          ------------------------------------------------------------    261

PcJBC1          FESGETYDLLFTDLIMPGGMNGVMLAREVKRRYPKIKVLLTTGYAESSIERTDLGGTEFE    500
PssB728a        FEAGATYDLLFTDLIMPGGMNGVMLAREVRRRFPKVKVLLTTGYAESSIERTDIGGSEFE    508
PstDC3000       FESGNMYDLLFTDLIMPGGMNGVMLAREVRRRYPKVKVLLTTGYAESSIERTDIGGSEFD    508
Xac306          LEKNPEVDALFTDLIMPGGMNGVMLAREARRMLPKIKILLTTGYADASIQRTDVGGAEFD    514
XccATCC33913    LEQHPEVDALFTDLIMPGGMNGVVLAREARRMLPKIKVLLTTGYADASIQRTDVGGAEFD    514
McbWSM1271      ------------------------------------------------------------    382
RltWSM2304      ------------------------------------------------------------    345
Bss168          ------------------------------------------------------------    261

PcJBC1          VVSKPCMPHDLARKVRQVLDGPNGVA    526
PssB728a        VVSKPCMPQDLARKVRQVLDGPNGIA    534
PstDC3000       VVSKPCMPHDLARKVRQVLDGPNGIA    534
Xac306          VVNKPYTQKELLKRIRMLLDGPTGVG    540
XccATCC33913    VVNKPYTQKELLKRIRMLLDGPTGVG    540
McbWSM1271      --------------------------    382
RltWSM2304      --------------------------    345
Bss168          --------------------------    261
```

Supplementary Figure S1. Multiple sequence alignment of the deduced LOV amino acid sequences. The deduced amino acid sequences of LOV proteins from *Pseudomonas cichorii* JBC1 (PcJBC1), *P. syringae* pv. *syringae* B728a (PssB728a), *P. syringae* pv. *tomato* DC3000 (PstDC3000), *Xanthomonas axonopodis* pv. *citri* 306 (Xac306), *X. campestris* pv. *campestris* str. ATCC 33913 (XccATCC33913), *Mesorhizobium ciceri* bv. *biserrulae* WSM1271 (McbWSM1271), *Rhizobium leguminosarum* bv. *trifolii* WSM2304 (RltWSM2304), and *Bacillus subtilis* subsp. *subtilis* 168 (Bss168) aligned with Clustal Omega. Light, Oxygen, or Voltage (LOV), histidine kinase (HK), response regulator (RR), and Sulphate Transporter and AntiSigma factor antagonist (STAS) domains are highlighted in blue, green, brown, and gray, respectively. An asterisk indicates complete residue conservation, a colon indicates strong group conservation, a period indicates weak group conservation, and a blank space indicates no conservation of residues.

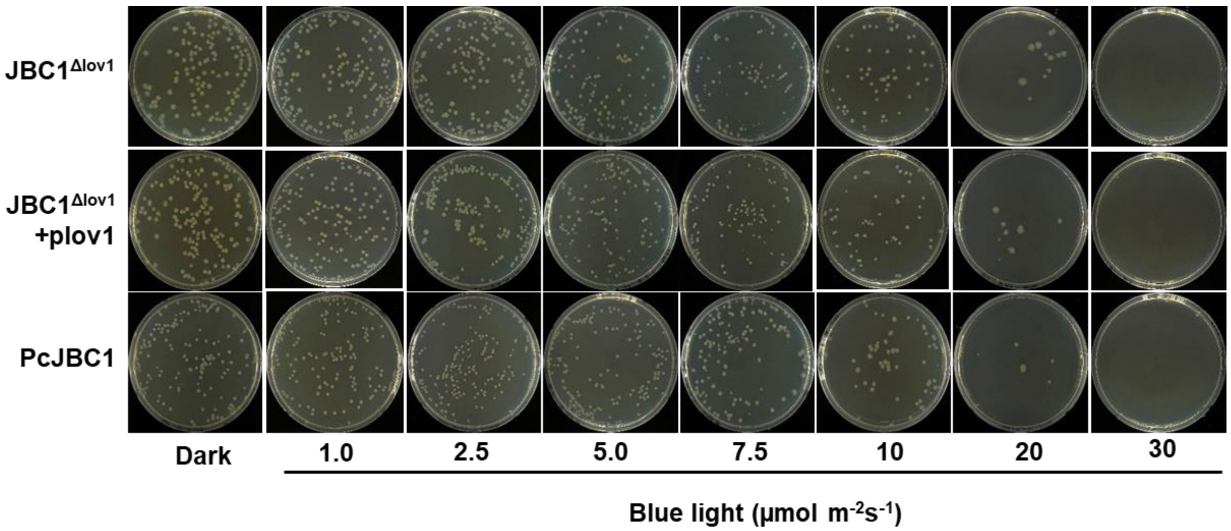

Supplementary Figure S2. Survival of *Pseudomonas cichorii* JBC1 cells after exposure to various intensities of blue light. The overnight cultures of each strain (PcJBC1, JBC1$^{\Delta lov1}$, and JBC1$^{\Delta lov1}$+plov1) were serially diluted and spread onto LB agar plates. The plates were incubated under blue light with various intensities (1, 2.5, 5.0, 7.5, 10, 20, and 30 μmol/m^{2}s) and dark conditions. The number of colonies was counted 48 h after incubation at 28°C.

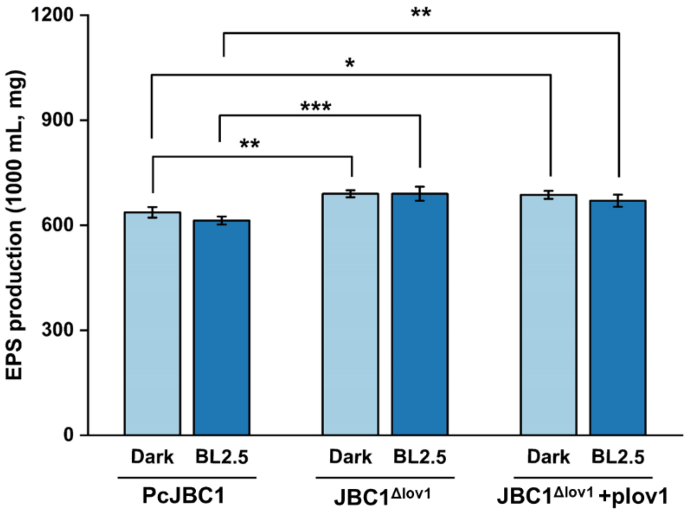

Supplementary Figure S3. Effects of Pc-LOV1 and blue light on exopolysaccharide production of *Pseudomonas cichorii* JBC1. PcJBC1, JBC1$^{\Delta lov1}$, and JBC1$^{\Delta lov1}$+plov1 were cultured in LB for 72 h under BL-illuminated (2.5 μmol/m^{2}s) and dark conditions. The cell-free supernatant obtained by centrifugation was supplemented with KCl and 95% ethanol and the precipitated crude EPS was harvested via centrifugation, dried overnight, and weighed. Each bar represents means ± standard deviation from two independent experiments in triplicate, and the p-values were indicated by asterisks, *p≤0.05, **p≤0.01, ***p≤0.001, according to Tukey's test.

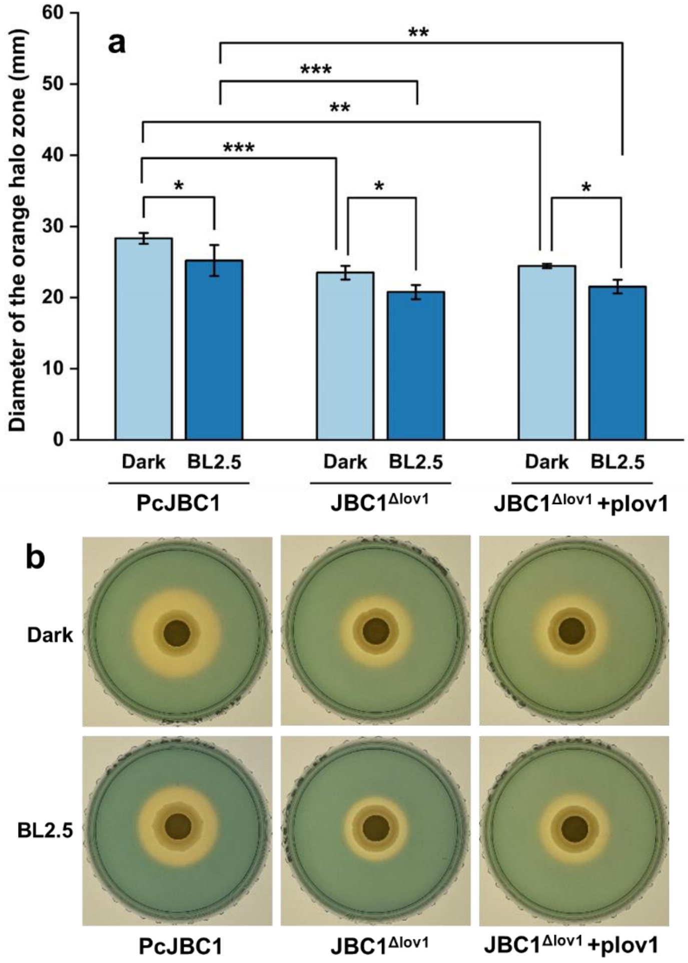

Supplementary Figure S4. Influence of Pc-LOV1 and blue light on siderophore production of *Pseudomonas cichorii* JBC1. (**a**) Overnight cultures of PcJBC1, JBC1$^{\Delta lov1}$ and JBC1$^{\Delta lov1}$+plov1 were spot-inoculated (20 μL) onto the sterile paper disks laid on the CAS plates, and the plates were incubated at 28°C for three days under blue light (2.5 μmol/m^{2}s) and dark conditions, and then diameter of orange halo zone was measured. Each bar represents means ± standard deviation from two independent experiments in triplicate, and the p-values were indicated by asterisks, *p≤0.05, **p≤0.01, ***p≤0.001, according to Tukey's test. (**b**) Orange halo zones on the CAS plates three days after incubation.

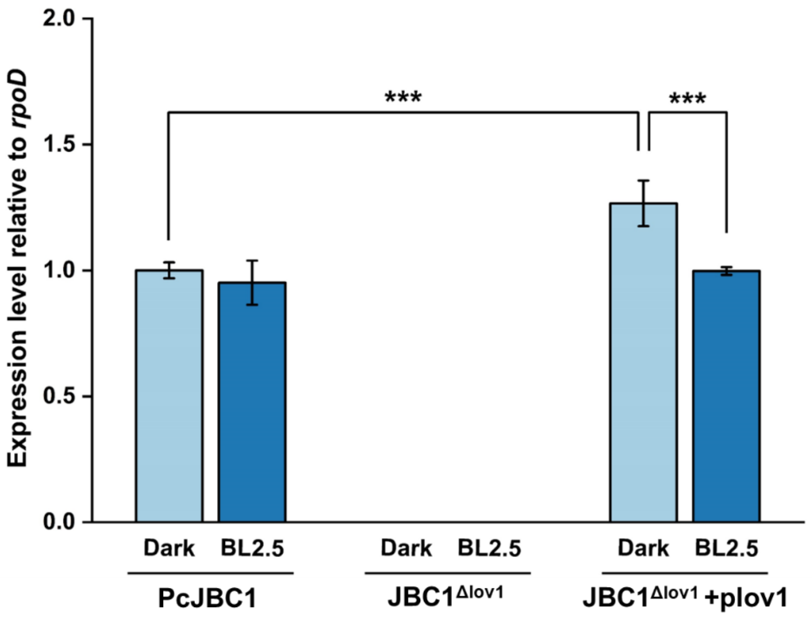

Supplementary Figure S5. Expression of *lov1* under the influence of blue light. The PcJBC1, JBC1$^{\Delta lov1}$, and JBC1$^{\Delta lov1}$+plov1 strains were cultured in minimal media under blue light and dark conditions. After 12 h of incubation, total RNA was isolated and qPCR analysis was performed for the expression of *lov1*. The housekeeping gene *rpoD* was used as an internal control. Fold differences were calculated using the ΔΔCT method with CT values obtained from qPCR and were normalized to the expression of genes in PcJBC1 grown in the dark. Expression values indicate average ± SD of three independent experiments, and the p-values were indicated by asterisks, $*p \leq 0.05$, $**p \leq 0.01$, $***p \leq 0.001$, according to Tukey's test.

Supplementary Table S1. Characteristics of Pc-LOV1-encoding genes identified in *Pseudomonas cichorii* JBC1 and other plant-associated bacteria

Species	Gene locus tag	Gene length (bp)	Identities[a]	MW (kDa)[b]	pI[b]	GRAVY[b]
PcJBC1	PCH70_11150	1581	526/526 (100%)	58.09	5.28	-0.174
PssB728a	Psyr_2700	1605	419/527 (80%)	59.02	5.35	-0.202
PstDC3000	PSPTO_2896	1605	419/527 (80%)	58.94	5.11	-0.205
Xac306	XAC2555	1623	313/519(60%)	58.92	5.30	-0.198
XccATCC33913	XCC2421	1623	320/523(61%)	59.02	5.24	-0.208
McbWSM1271	Mesci_3196	1149	96/216(44%)	42.79	5.67	-0.461
RltWSM2304	Rleg2_5263	1038	73/162(45%)	37.91	8.56	-0.154
Bss168	BSU_30340	786	53/130(41%)	29.19	4.88	-0.140

[a]The deduced amino acid sequence of the Pc-LOV1 protein of *Pseudomonas cichorii* JBC1 (PcJBC1) was compared to that of *P. syringae* pv. *syringae* B728a (PssB728a), *P. syringae* pv. *tomato* DC3000 (PstDC3000), *Xanthomonas axonopodis* pv. *citri* 306 (Xac306), *X. campestris* pv. *campestris* str. ATCC 33913 (XccATCC33913) and *Mesorhizobium ciceri* bv. *biserrulae* WSM1271 (McbWSM1271) and *Rhizobium leguminosarum* bv. *trifolii* WSM2304 (RltWSM2304) and *Bacillus subtilis* Ytva (BsYtva) using ClustalW2.

[b]The Molecular weight (MW, kDa), isoelectric point (pI), and grand average of hydropathy value (GRAVY) were predicted using ExPaSy-ProtParam.

Supplementary Tables S2. Primers used in this study

Name	Primer Sequence (5′→3′)[a]	Use
sgLOV1-F	GTGGGCGTTTCGATGAACACCCGA	Generation of target sequence to edit *lov1*
sgLOV1-R	AAACTCGGGTGTTCATCGAAACGC	
R-Amp	CAAGGCGAGTTACATGATCCCCCA	Checking presence of pACRISPR-sgRNA-*lov1*
LOV1-Us-F	TTTTGAGATCTGTCCATACCCATGGTCTAGAGGTGGCCCCCCAACCCAGAC	Amplification of the upstream region of the *lov1* gene (500 bp)
LOV1-Us-R	CTGTTGCACGTAGCCGCAACAGGCACTGTCACCCGGACTTTACTATCGCTCACACACCC	
LOV1-Ds-F	GGTGGGTGTGTGAGCGATAGTAAAGTCCGGGTGACAGTGCCTGTTGCGGCTAC	Amplification of the downstream region of the *lov1* gene (500 bp)
LOV1-Ds-R	TCTGAATGGCGGGAGTATGAAAAGTCTCGAGTAGGCACTGCTTGAACAACAACGCAC	
LOV1-HindIII-500Us-F	AGTGACAAGCTTGGTGGCCCCCCAACCCAG	Clone *lov1* gene including its promoter regions into pUCP18 vector
LOV1-BamHI-R	ACCCGTGGATCCTCAGGCTACGCCGTTGG	
BamHI-Lov1-1F	CTAGGATCCGTGAATAACCGGGTAACGG	To clone *lov1* gene into pET28a vector
HindIII-Lov1-1581R	CATGAAGCTTTCAGGCTACGCCGTTG	
T7-Promoter-F	GCGAAATTAATACGACTCACTATAGGG	To confirm recombinant plasmid pET28a_*lov1*
T7-Terminator-R	GTTATGCTAGTTATTGCTCAGCGG	
Lov1-F	TGTCCCTCGCATCAAACGAA	RT-PCR for *lov1*
Lov1-R	TTCTGCACCGAAAGTACGCT	
FliA-F	TGAAACCTATGCCGGTATCC	RT-PCR for *fliA*
FliA-R	CTTAACTGGAGTTCGGCAGC	
FliG_F	TGTCGGTGCAGATCGAAGAC	RT-PCR for *fliG*
FliG_R	TTTCCTTGATCGCCTCGTCC	
FlgJ_F	CGGCTGGGGTAAATCCATCA	RT-PCR for *fliJ*

FlgJ_R	CTCCTTGACCATCTTGCCGT	
CifA-F	CCTATGCCGAATCGATGCCT	RT-PCR for *cifA*
CifA-R	CATCCAGTGGTGGTCTTCCC	
CifB-F	CTTTGCCACCACCAACGAAG	RT-PCR for *cifB*
CifB-R	ACGGTTCAGATAACCCTGCG	
HrpA-F	CTGATGGACTCTACTGCTGCTG	RT-PCR for *hrpA*
HrpA-R	CGTTGATGGTATCTGTCTGCAT	
HrpL-F	CAGCGAGAAAAAGCTGCGAG	RT-PCR for *hrpL*
HrpL-R	GATTGGCTGCAATCCCGAAC	

[a] Restriction enzyme sites are underlined

Supplementary Table S3. Bacterial strains and plasmids used in this study

Strain or plasmid	Description and/or relevant genotype or phenotype	Reference or source
Strains		
PcJBC1	*Pseudomonas cichorii* JBC1, wild type	Ramkumar et al. (2015)
JBC1$^{\Delta lov1}$	*lov1* – deletion mutant of PcJBC1	This study
JBC1$^{\Delta lov1}$+plov1	Complementary strains JBC1$^{\Delta lov1}$ contains pUCP18-*lov1*	This study
PcJBC1-pCasPA	PcJBC1 contains pCasPA plasmid	This study
E. coli TOP10 PACrispr-SgRNA^{lov1}	*E. coli* TOP10 contains PACrispr-SgRNA^{lov1}	This study
E. coli TOP10 PACrispr-SgRNAlov1-Us-Ds	*E. coli* TOP10 contains PACrispr-SgRNA^{lov1}-Us-Ds	This study
E. coli TOP10-*lov1*	*E. coli* TOP10 contains recombinant plasmid pET28a-*lov1,* Kanamycin	This study
E. coli BL21-*lov1*	*E. coli* BL21 contains recombinant plasmid pET28a-*lov1,* Kanamycin	This study
Plasmids		
pCasPA	pCasPA plasmid, Tetracyclin	This study
PACrispr	PACrispr plasmid, Ampicillin	This study
PACrispr-SgRNA^{lov1}	PACrispr with single guide of *lov1* gene, Ampicillin	This study
PACrispr-SgRNA^{lov1}-Us-Ds	PACrispr-SgRNA^{lov1} with upstream and downstream fragments of *lov1* gene, Ampicillin	This study
pUCP18	pUCP18 vector, Ampicillin	This study
pUCP18-*lov1*	pUCP18 with *lov1* genes and its natural promoter, Ampicillin	This study
pET28a	pET28a vector, Kanamycin	This study
pET28a-*lov1*	pET28a with *lov1* gene, Kanamycin	This study
